# Systematic review of mHealth and digital health interventions to improve childhood vaccination uptake in 19 Sub-Saharan African countries

**DOI:** 10.1371/journal.pone.0324117

**Published:** 2025-12-23

**Authors:** Alex Bhattacharya, Chidera Mark-Uchendu, Christa Hansen, Jay Evans

**Affiliations:** 1 University of Edinburgh, Usher Institute of Population Health Sciences and Informatics, Edinburgh, United Kingdom; 2 Imperial College London, Institute of Global Health Innovation, London, United Kingdom; 3 Imperial College London, Centre for Health Economics and Policy Innovation, London, United Kingdom; 4 University College London, Centre for Teaching and Learning in Economics, London, United Kingdom; King Faisal University, SAUDI ARABIA

## Abstract

Mobile health and digital health (mHealth/DH) interventions have been shown to support immunisation programmes in Sub-Saharan Africa (SSA) and improve uptake of life-saving vaccines. As 19 SSA countries were targeted to begin rolling out the two new malaria vaccines (RTS,S/AS01 and R21/Matrix-M) in 2024, this systematic review aims to investigate which mHealth/DH interventions are most effective at increasing vaccination uptake (by assessing vaccination coverage and timeliness outcomes) in these countries. The review assessed the effectiveness of mHealth/DH interventions for increasing uptake of Diphtheria–Tetanus–Pertussis or Pentavalent vaccines (DTP/Pentavalent). As with any multi-dose vaccine, the DTP/Pentavalent vaccine requires multiple doses to ensure its maximum protective benefit, therefore maintaining schedule adherence and ensuring its timely completion is essential. Thus, identifying strategies to support adherence, such as digital appointment reminders, remains a public health priority. Eight electronic databases were searched, alongside selected grey literature sources. A narrative synthesis was conducted with studies grouped by mHealth/DH intervention-type. Included studies were assessed for risk of bias using RoB2 and ROBINS-I, and certainty of evidence was evaluated using the GRADE approach. 14 studies were included, comprising both randomised and non-randomised control trials. However, only 4 out of the 19 SSA countries were represented (Nigeria, Kenya, Burkina Faso and Cote D’Ivoire). All interventions investigated were appointment reminders. Generally, all intervention-types were positively associated with vaccination coverage and timeliness. SMS-based interventions showed modest effects, whereas interventions incorporating voice components (phone calls/voice messages) tended to yield larger effects. The certainty of evidence ranged from very low to moderate depending on the intervention-type and outcome pairing. The findings offer evidence-based insights to guide the development and implementation of mHealth/DH interventions within SSA childhood immunisation programmes. While interventions with voice-based components appear particularly promising, the limited certainty of evidence demonstrates further high-quality, context-specific research is required to draw stronger conclusions.

## Introduction

2024 was a monumental year for public health and the fight against malaria. The World Health Organization (WHO) has now approved two malaria vaccines for use (RTS,S/AS01 and R21/Matrix-M), and these are beginning to be rolled out in 19 countries (Benin, Burkina Faso, Burundi, Cameroon, Central African Republic, Chad, Cote d’Ivoire, Democratic Republic of Congo, Ghana, Guinea, Kenya, Liberia, Malawi, Mozambique, Niger, Nigeria, Sierra Leone, South Sudan and Uganda) across Sub-Saharan Africa (SSA) [1]. This raises important questions about how best to support childhood vaccination uptake in this context.

There is increasing evidence to suggest that mobile health (mHealth) and digital health (DH) interventions could optimise immunisation programmes and improve uptake of lifesaving vaccines [2,3]. Whilst no studies have yet assessed mHealth or digital health (mHealth/DH) interventions for malaria vaccine uptake directly, examining interventions used to improve uptake of other multi-dose routine childhood vaccines (such as the Diphtheria–Tetanus–Pertussis or Pentavalent (DTP/Pentavalent) vaccines) can offer relevant insights that may inform future vaccination programme design in similar contexts. Therefore, this systematic review investigates mHealth/DH interventions for increasing DTP/Pentavalent vaccination uptake in these 19 SSA countries. Although this review focuses on mHealth/DH interventions for improving DTP/Pentavalent vaccine uptake, the 19 countries were selected because of their planned malaria vaccine rollouts, which provided the contextual motivation for the study.

mHealth is an emerging field that involves the use of devices such as mobile phones and other wireless mobile devices to enhance medical and public health practices, and improve healthcare delivery, patient outcomes and health system efficiency [4,5]. mHealth interventions range from simple phone calls and short message service (SMS) messaging to more complex technologies like mobile applications, mobile data collection platforms and wireless data transmissions [6]. These support various activities, including facilitating health-related communications, remote disease surveillance, health education, data collection and analysis, healthcare worker (HCW) assistance, teleconsultations, research activities and streamlined patient management [5,7,8]. In contrast, digital health (DH) is a broad umbrella term encompassing eHealth (electronic health), which includes mHealth, in addition to areas such as ‘big data’ computing, genomics and artificial intelligence (AI) [9]. DH involves a wider range of technology, including electronic health records (EHR), telemedicine, online health education platforms and health information systems (such as the widely used District Health Information System2 (DHIS2)) [10]. These tools enable streamlined data collection, facilitating data-driven healthcare delivery decision-making [11].

As healthcare infrastructure is limited in certain SSA regions, mHealth/DH can support immunisation programmes where mobile phone penetration is high [12,13]. Examples of immunisation-related mHealth interventions include SMS reminders to reduce missed appointments and ensure timely follow-ups, mobile data collection, and mHealth educational platforms to inform individuals and address vaccine hesitancy [12]. Other DH tools are also useful in SSA immunisation programmes, including digital data management and analysis tools, tracking vaccination coverage, supply chain management, and telemedicine platforms for remote consultations or vaccinator training [3,14].

As the WHO targeted 19 SSA countries for malaria vaccine rollout in 2024, identifying strategies that have supported uptake of other childhood vaccines in this same context is of clear public health interest [1]. Investigating strategies and interventions used in similar contexts is well established as a key precursor in developing effective interventions [15]. Thus, this review aims to contribute to the evidence base on the use of mHealth/DH interventions for increasing childhood vaccination uptake in SSA.

Vaccination is a well-established tool for disease prevention and reducing mortality due to vaccine-preventable diseases (VPDs) [16]. Since its launch in 1974, the WHO’s Expanded Programme on Immunization (EPI) has been instrumental in improving childhood vaccination coverage globally, particularly in LMICs, including those in SSA [17]. Immunisations currently prevent an estimated 3.5–5 million deaths annually from diseases such as diphtheria, tetanus, pertussis, and measles [16].

The DTP/Pentavalent vaccine is important for preventing serious and life-threatening diseases in young children. The DTP vaccine protects against Diphtheria, Tetanus and Pertussis, whereas the Pentavalent vaccine targets those diseases plus Hepatitis B and Haemophilus influenzae type b (HiB)) [18]. The DTP/Pentavalent vaccine schedules are well established within the EPI and are largely standardised across countries, being delivered at 6, 10, and 14 weeks of age [19,20]. By studying interventions aimed at increasing uptake of DTP/Pentavalent vaccines, this review provides evidence of interventions aimed at supporting vaccination uptake in a child’s first months of life. Furthermore, DTP/Pentavalent coverage is widely used as a key indicator of immunisation programme performance and health system robustness [21]. This established role makes it a practical choice for informing intervention implementation research, and interventions targeting DTP/Pentavalent vaccine may have transferability across childhood vaccination programmes in similar settings.

Multi-dose vaccines such as the DTP/Pentavalent vaccines require multiple appointments, which can pose challenges for maintaining schedule adherence and ensuring the schedule’s timely completion. These multi-dose vaccine schedules often result in dropouts or delays, limiting a vaccine’s protective benefit [22]. Therefore, it is essential to identify effective strategies to support schedule adherence, such as the use of digital reminder systems. Our review investigates the effects of implementing mHealth/DH interventions on DTP/Pentavalent vaccination uptake to generate evidence-based insights into their use in future childhood immunisation programmes. However, consideration of context-specific factors is essential if applying these findings to other contexts.

Challenges facing SSA immunisation programmes are well documented and relate to socio-economic, infrastructural and political factors [23]. Examples include: inadequate healthcare infrastructure presenting logistical challenges which disrupt vaccine cold chain storage and their distribution and uptake [24], supply chain issues which can result in vaccine wastage or ‘stockouts’ resulting in unavailability of required vaccines [25,26], and vaccine hesitancy due to misinformation or cultural beliefs [27]. Economic constraints are also a key barrier, with many SSA countries relying on external funding to execute national immunisation programmes [23]. On an individual level, poor adherence to immunisation appointments means essential immunisation schedules are not completed, reducing efficacy and life-saving benefits of the vaccines [22,28]. Although some of these challenges are beyond the scope of mHealth/DH interventions, these technologies can offer solutions for poor adherence in appointments, vaccine hesitancy and enhancing supply chain management [12,29,30].

Although mobile phone affordability is a barrier for many in SSA, huge projected growth in SSA mobile phone use over the coming decades presents a significant opportunity for mHealth/DH where healthcare infrastructure may be lacking [31]. Despite this, equity considerations relating to the ‘digital divide’ are essential during intervention development [32].

Initial scoping searches of mHealth/DH interventions for increasing vaccination uptake identified four categories for immunisation programme optimisation. These are: communication technology (such as SMS appointment reminders), stock management mobile applications, surveillance or data analysis tools and electronic immunisation registries (EIR) [12,33–35]. Leveraging these technologies could be hugely advantageous for improving immunisation programmes and related health outcomes. Most published experimental literature related to mHealth/DH and communication technology focused on appointment scheduling and reminders.

Several systematic reviews on similar topics were discovered [12,33,36]. However, this review will seek to answer a different research question by focusing specifically on mHealth/DH interventions targeting uptake of multi-dose childhood vaccines, namely DTP/Pentavalent, in the 19 SSA countries where the malaria vaccines are being introduced. This geographic and programmatic focus offers a distinct contribution to the evidence base. The review also searches for both mHealth and DH interventions. Additionally, given the rapid evolution of mHealth/DH tools and growing body of implementation research in LMICs, regular evidence synthesis is warranted to identify the most promising approaches and update the evidence base [37]. Findings from this review aim to inform context-specific design and implementation of mHealth/DH interventions in childhood vaccination programmes across SSA.

The review will seek to answer the research question: ‘Which mHealth or digital health interventions have proved most effective in increasing childhood vaccination uptake (of DTP/Pentavalent vaccine) in the 19 SSA countries rolling out the malaria vaccines in 2024?’, by addressing three outlined objectives: **(1)** Identify current up-to-date (as of January 2025) evidence of mHealth/DH interventions for increasing vaccination uptake and coverage of DTP/Pentavalent vaccines in the 19 SSA countries of interest; **(2)** Report on any other factors from included studies relating to uptake of childhood immunisations, such as timeliness of vaccination, missed opportunities for vaccination, or vaccine wastage in the selected countries; and **(3)** Provide strategic, evidence-based insights, grounded in the review’s findings, on how mHealth/DH interventions can support improved vaccination uptake. While synthesising evidence from DTP/Pentavalent vaccination is the primary focus, findings may offer some valuable transferable insights for ongoing malaria vaccine implementation. Additional implementation challenges, risks, or mitigating factors will also be considered.

## Methods

This systematic review of quantitative studies was guided by the Cochrane Methodology and followed the PRISMA reporting guidelines [38,39]. The completed PRISMA checklist is included in S1 File. Initial scoping searches to assess volume of literature on the topic were conducted on Google Scholar and PubMed. The review was conducted in line with a predefined protocol which was registered on PROSPERO in October 2024 (CRD42024587428).

The PICOS (Population, Intervention, Comparison, Outcome and Study Design) framework was used to structure several review stages, including the search strategy, inclusion/exclusion criteria, study selection process and data extraction [40]. We adapted this framework to include an additional ‘S’ representing *Setting* given the review’s specific geographic context. The resulting PICOSS framework is shown in S2 File. The outcomes of interest were selected to provide a comprehensive understanding of mHealth/DH interventions’ effect on vaccination uptake. The review investigated both key parameters relating to vaccination uptake: *coverage* (indicating the proportion of study population under investigation that received DTP/Pentavalent vaccine), and *timeliness* (indicating whether vaccines were administered at recommended times ensuring optimal effectiveness) [41].

While vaccination coverage is a simpler concept and straightforward to assess, timeliness is a more complex aspect of vaccination programmes [41,42]. In this review, timeliness was defined as whether the DTP/Pentavalent vaccines were administered within the recommended age intervals specified by each study country’s national immunisation schedules (typically 6, 10, and 14 weeks). Extracted outcome data were categorised as either ‘Coverage’ or ‘Timeliness’. In many cases this followed terminology reported by study authors, for example, ‘DTP3 coverage (%)’, or ‘Timeliness of receipt (%) (within two-weeks of recommended schedule)’. Where outcome measures were ambiguous or overlapping, decisions were made through consensus among reviewers (AB and CMU) on whether reported outcome should be categorised as ‘Coverage’ or ‘Timeliness’.

Eight electronic databases were selected based on their relevance for global health research. These were MEDLINE (Ovid), Global Health (Ovid), Embase (Ovid), Scopus, Web of Science, Cochrane CENTRAL, WHO’s African Index Medicus (AIM) and African Journals Online (AJOL). The search strategy development was done in collaboration with University of Edinburgh’s College of Medicine and Veterinary Medicine’s Academic Support Librarian. The search strategy for each database is shown in S3 File. Final searches were conducted on January 17th, 2025. A targeted grey literature search was conducted using OpenHIA [43], OpenMRS [44], and WHO’s mHealth/DH working group publications [45], to identify relevant unpublished studies and reduce the risk of publication bias. This was not exhaustive due to resource constraints. The inclusion of a grey literature search aimed to minimise publication bias; however, we recognise the limited scope of the search may have introduced bias through the underrepresentation of relevant unpublished studies or country-specific reports. Additionally, to help reduce the risk of missing key studies, we also screened the reference lists of similar reviews [12,33,36].

All retrieved studies were imported into Covidence review management software which was used to facilitate the review process. Duplicate records were automatically identified and removed on Covidence during the import process. The resulting records were then manually reviewed to ensure accuracy in duplicate removal. A PICOSS-based inclusion and exclusion criteria guided the screening process (S2 File). AB and CMU conducted title and abstract, and full-text screening, independently, with JE resolving any arising conflicts. Study selection was presented using an adapted PRISMA flowchart [39]. As experimental epidemiological studies (RCTs and non-RCTs) are the gold standard for testing intervention effectiveness, therefore only these study types were included [46]. Data extraction was also conducted by two researchers independently (AB and CMU). AB and CMU conducted an independent risk of bias assessment of all included studies to assess each included study’s methodological rigour. Cochrane’s Risk of Bias2 (RoB2) tool was used for assessing RCTs [47], whilst Cochrane’s Risk of Bias in Non-randomised Studies (ROBINS-I) tool was used to assess non-RCTs [48]. Additionally, a discrete certainty of evidence assessment was conducted to assess confidence in the review’s findings for each outcome (mHealth/DH intervention effect on: (1) vaccination coverage and (2) vaccination timeliness) using the GRADE (Grading of Recommendations Assessment, Development and Evaluation) framework [49,50].

### Data analysis and narrative synthesis

As this review aimed to identify which interventions were most effective at increasing vaccination uptake in the specific context, the analysis aimed to evaluate the relative effectiveness of each included mHealth/DH intervention on vaccination coverage and timeliness. AB and CH conducted an assessment of the extracted data and made a decision on synthesis approach. Due to extremely high heterogeneity reported (**Fig 3** and **4**), a narrative synthesis guided by synthesis without meta-analysis (SWiM) reporting guidelines was conducted [51]. This approach was further supported by very high heterogeneity reported in similar meta-analysis studies [33,36]. For the narrative synthesis, studies were grouped together by mHealth/DH intervention-type. These intervention-type groups were: ‘SMS-Only’, ‘SMS-Plus’, ‘SMS and/or Voice Messages or Phone calls’, ‘Phone calls only’, and ‘Electronic Immunisation Alert Wristband’. A description of each group and the studies included in each is shown in S4 File. The synthesis method entailed a description of positive or negative associations, a consideration of point estimate precision, and each study’s risk of bias. Additionally, for narrative synthesis best practice suggests that syntheses are accompanied by discrete certainty of evidence assessment [49–51]. As mentioned, the certainty of the evidence for each intervention-outcome pairing using the GRADE framework was conducted. This considered five domains: risk of bias, inconsistency, indirectness, imprecision, and publication bias. AB and CMU independently conducted the assessments, resolving any discrepancies through discussion. Final certainty ratings were classified as high, moderate, low, or very low. Due to a lack of variation in results due to study design, it was deemed appropriate to analyse findings from both RCT and non-RCT study designs together.

**Fig 1:**
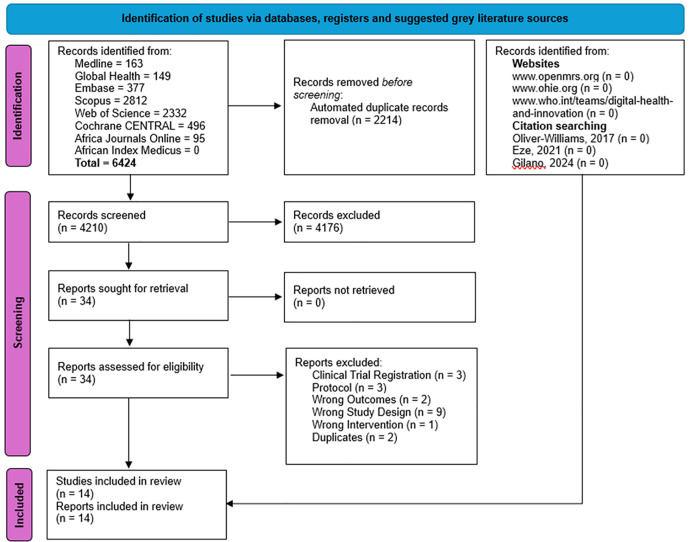
PRISMA flow chart outlining the study selection process for this systematic review. Adapted from Page *et al* (2021) [39].

**Fig 2:**
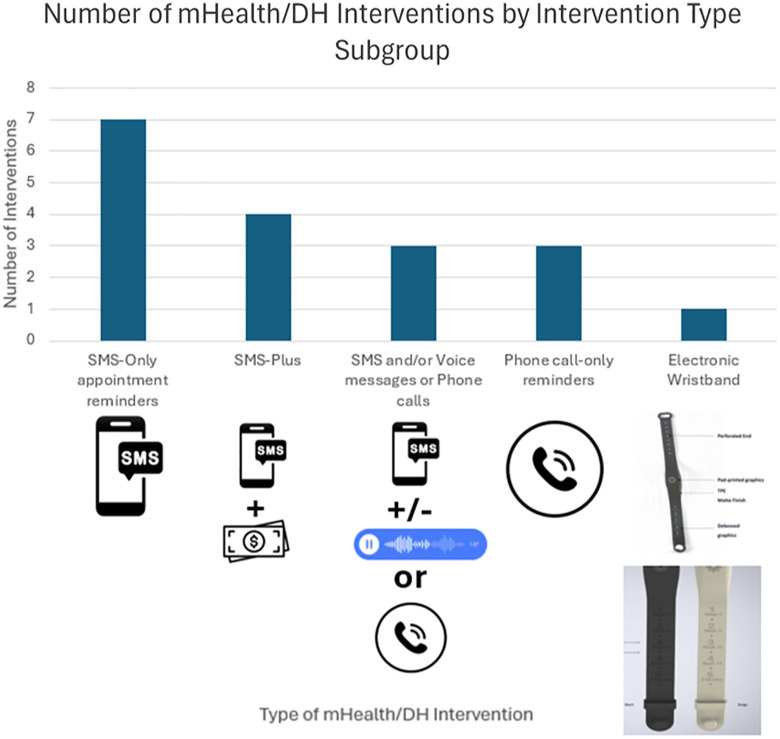
The number of interventions investigated in this review by intervention-type subgroup and pictograms of each intervention. Note that individual interventions in multiple-arm RCT and non-RCTs have been investigated separately. (Created on Microsoft Excel and PowerPoint, pictures taken from canva.com and Sampson *et al* (2023) [64]).

Forest plots were created using Jamovi statistical software to provide visual representation of the review’s quantitative findings and to support the narrative synthesis. These only presented data on the third DTP/Pentavalent dose (DTP/Pentavalent3). Using the final dose provided a robust indicator of mHealth/DH intervention effectiveness on vaccine coverage and timeliness, and indicated whether the vaccination schedule was completed, thus allowing assessment of intervention effectiveness on schedule adherence. Each included intervention was analysed individually, therefore multi-arm studies provided multiple point estimates, allowing a visual comparison of all included interventions. It should be noted that each intervention from the multi-arm RCTs or non-RCTs was compared against the same control, however, this does not bias the results as pooled estimates were not calculated.

The standardised synthesis metric was logarithm of the odds ratio (logOR), which provided information on associations between mHealth/DH intervention and outcome (vaccination coverage or timeliness). All values were transformed to logOR, as this metric provided a more robust visualisation of strength and certainty of reported associations [52,53]. LogORs were calculated from the data extracted from included studies, the raw data used to calculate point estimates is shown in S6 File. 95% confidence intervals were provided for each point estimate, along with heterogeneity statistics (including I^2^).

### Ethics approval

No primary data were collected for this study. Ethical approval was therefore sought and granted by the University of Edinburgh’s Usher Masters’ Research Ethics Group (UMREG).

## Results

The PRISMA flow chart outlines the study selection process (Fig 1). The full search identified 6424 records, however, following full-text screening only 14 studies met the inclusion criteria and were included in the study [54–67]. The full list of included studies is shown in S4 File.

### Study characteristics

Table 1 summarises the characteristics of the 14 included studies [54–67]. Ten of the included studies were RCTs [54–60,62,65,67], with the remaining four studies comprising non-RCTs [61,63,64,66]. The majority of studies were conducted in West Africa (12 studies) [54–58,61–67], and only 4 of the 19 SSA countries targeted for malaria vaccine rollout (Nigeria [54,55,57,58,61–64,66,67], Burkina Faso [65], Cote D’Ivoire [56] and Kenya [59,60]) were represented in the study. All the interventions included related to appointment reminders to encourage and remind mothers/caregivers to bring their children to immunisation clinics to receive DTP/Pentavalent doses. As each intervention was analysed independently, the number of mHealth/DH interventions and the type of interventions investigated in this review is shown in Fig 2.

**Table 1:** Study characteristics of the 14 included studies.

Author and Year	Country (Region)	Urban or Rural Setting	Study Design	mHealth/DH Intervention	Intervention Details	Control Details	Study Population	Follow Up Length	Outcomes Measured (Coverage or Timeliness)	DTP or Pentavalent Vaccine	Additional Notes
Brown and Oluwatosin, 2017 [54].	Nigeria (West Africa)	Urban	RCT (2-arm) (Clustered design)	‘Phone call-only’ appointment reminders	Intervention group received 1x phone call reminder 2days before their child’s next immunisation appointment. If appointment was missed, then ‘recall’ phone call was made.	Usual care – no phone call reminders	595 mother/infant pairs (295 in the intervention group and 300 in the control group). Children were aged 0–12weeks at recruitment.	13 months	Immunisation compliance rate (Considers the required number of doses at appropriate age and recommended intervals [schedule adherence/timeliness]) (%). **Categorised as coverage.**	DTP vaccine	Used same study population as Brown *et al* (2016). Group **A** + **C** in Brown *et al* (2016) = Intervention group. Group **B** + **D** in Brown *et al* (2016) = Control. RCT registered and results analysed retrospectively.
Brown *et al*, 2016 [55].	Nigeria (West Africa)	Urban	RCT (4-arm) (Clustered Design)	‘Phone call-only’ appointment reminders	**Group A** received phone calls 2days before and 1day before the child’s immunisation appointment. ‘Recall’ phone calls were made if an appointment was missed. **Group B** no mHealth/DH intervention so not included. **Group C** received both phone call reminders and vaccinator/HCW training on immunisation theory.	**Group D** received usual care – no intervention	595 mother/infant pairs total study population (295 in the intervention groups [148 in **A** and 147 in **C**] and 150 in the control group [**D**]. **Group B** not included. Children were aged 0–12weeks at recruitment.	12 months	Immunisation schedule completed or not completed (All essential immunisations including 3x doses of DTP/Pentavalent) (%, RR and OR). **Categorised as coverage.**	DTP vaccine	This report is the larger study mentioned above in Brown and Oluwatosin (2017)
Dissieka *et al*, 2019 [56].	Cote D’Ivoire (West Africa)	Both	RCT (2-arm) (Individual)	Automated SMS or Voice message appointment reminders	Intervention group received either SMS or voice message prior to each scheduled immunisation visit. 2x additional reminders were sent if appointment was missed. Reminders were automatically sent out. Mothers could choose whether they received SMS or phone call, and language of reminders.	Usual care – no intervention	1596 mother/infant pairs (798 in intervention group and 798 in the control group). 736 were from Urban setting, 560 were from Semi-urban setting, 300 were from Rural setting).	12 months	Attendance at each of the 3x Pentavalent vaccination appointment (% and aOR). **Categorised as coverage.**	Pentavalent vaccine	
Ekhaguere *et al*, 2019 [57].	Nigeria (West Africa)	Semi-rural	RCT (2-arm) (Individual)	Automated SMS and Voice message appointment reminders	Intervention group received automated phone call and text messaging appointment reminders, sent out 2days, and 1day, before the scheduled appointment for 3x Pentavalent doses. Text messages were in English, whereas phone calls were in English and Yoruba.	Usual care – no intervention. Child immunisation card listed when child was to receive immunisations.	600 mother/infant pairs (300 in the intervention group and 300 in the control group).	12 months	Proportion of infants that received Pentavalent dose1, dose 2 and dose 3 (% and RR). **Categorised as coverage.**	Pentavalent vaccine	
Eze and Adeleye, 2015 [58].	Nigeria (West Africa)	Urban	RCT (2-arm) (Individual)	‘SMS-only’ appointment reminders	Intervention group was sent SMS appointment reminder messages 1day before the child’s next immunisation appointment. ‘Recall’ SMS messages were sent 1day before the next immunisation session when an appointment was missed.	Usual care – no intervention	1001 mother/infant pairs (500 in intervention group and 501 in control group, 96 were lost to follow up so 905 were included in analysis (452 in intervention group and 453 in control group).	18 weeks	DTP3 Coverage (%) and Timeliness of receipt (%). **Both coverage and timeliness.**	DTP vaccine	
Gibson *et al*, 2017 [59].	Kenya (East Africa)	Rural	RCT (4-arm) (Clustered design)	Automated SMS reminders and SMS plus cash incentives (‘SMS-Plus’)	**Group A** received only SMS appointment reminders. **Group B** received SMS appointment reminders + cash incentive of 75KES, **Group C** received SMS appointment reminders + cash incentive of 200KES. All reminders were sent 1day before scheduled immunisation appointment (6weeks, 10weeks, 14weeks for 3 pentavalent doses).	**Group D**. Usual care – no SMS reminders or financial incentives.	2018 mother/infant pairs (489 in the control group, 476 in SMS only group, 562 SMS + 75KES group and 491 SMS + 200KES group).	12 months	Proportion of fully immunised children at 12months (included 3x Pentavalent doses plus BCG, 3x OPV and MCV) (% and RR) and timeliness of receipt (within 2weeks of recommended schedule) (% and RR). **Both coverage and timeliness.**	Pentavalent vaccine	
Haji *et al*, 2016 [60].	Kenya (East Africa)	Both	RCT (3-arm) (Clustered design)	Automated ‘SMS-only’ appointment reminders	Intervention group received 2x SMS appointment reminders 2days before child’s next appointment for 2nd and 3rd DTP doses. The 1st message reminded caretaker of the next due date of appointment and where to attend. The second message reminded caretakers that appointment was that day. Messages were in both Kiswahili and English.	Usual care – non-SMS. Appointment was recorded in child’s health book.	1116 mother/infant pairs (372 in SMS intervention group, 372 in Sticker reminder group [Non-mHealth/DH intervention so not included] and 372 in control group).	14 weeks	Penta2 and Penta3 coverage (%), and dropout rate (%). **Categorised as coverage.**	Pentavalent vaccine	Another non-mHealth/DH intervention was included in this study of ‘sticker reminders’, however, was not included in this study.
Ibraheem *et al*, 2021 [61].	Nigeria (West Africa)	Urban	Non-RCT (4-arm) (Clustered design)	Phone call appointment reminders (**A**), ‘SMS-only’ appointment reminders (**B**), or SMS health education (‘SMS-Plus’) (**C**)	**Group A** received phone call appointment reminders. **Group B** received SMS text message appointment reminders. **Group C** received health education SMS messages. **Group A** and **B** received reminders day before scheduled appointment. Phone call reminders were in English and/or Yoruba. SMS health education messages were sent at week5, week9 and 8months.	**Group D** received usual care – no intervention	560 mother/infant pairs (140 in **Group A** [phone call reminder group], 140 in **Group B** [SMS reminder intervention group], 140 in **Group C** [SMS health education intervention group], 140 in **Group D** [control group]).	9 months	Vaccination coverage (%), proportion of infants that received timely Penta1, Penta2 and Penta3 (%) and likelihood of presentation for appointment (OR). **Both coverage and timeliness.**	Pentavalent vaccine	All intervention subgroups represented by this study
Kawakatsu *et al*, 2020 [62].	Nigeria (West Africa)	Urban	RCT (2-arm) (Individual)	Automated ‘SMS-only’ appointment reminders	Intervention group received SMS appointment reminders in English, 2days before an upcoming appointment. In the event of a no show, an additional SMS reminder was sent 7days after the original appointment date.	Usual care – no intervention	8337 mother/infant pairs attending appointments at 31 primary healthcare centres, 4893 were in intervention group and 3444 were in control group.	3 months	Return rate for subsequent immunisation appointment (% and aOR). **Categorised as timeliness.**	Pentavalent vaccine	
Oladepo *et al*, 2020 [63].	Nigeria (West Africa)	Rural	Non-RCT (2-arm) (Clustered design)	Automated SMS immunisation education/reminders (‘SMS-Plus’)	SMS immunisation education messages sent 3x/week for 10months. Messages focused on importance of keeping to immunisation appointment, benefits of keeping routine appointments, benefits of timely and full completion of all basic essential immunisations and consequences of refusal/non-completion. Messages were in English but also translated in Yoruba, Ijaw, Hausa and Igbo.	Usual care – no intervention. However, were given flyers on the importance of adequate child nutrition and growth monitoring.	3500 mother/infant pairs (1750 in intervention group and 1750 in control group).	10 months	Completion of all immunisations (%) and coverage of Penta1, Penta2 and Penta3 (%). **Categorised as coverage.**	Pentavalent vaccine	
Sampson *et al*, 2023 [64].	Nigeria (West Africa)	Both	Non-RCT (2-arm) (Clustered design)	Wearable electronic immunisation appointment alert wristband	The electronic immunisation alert wristband was worn by caregiver and programmed to flash a red light in the lead up to the child’s next immunisation appointment and to the day of appointment. The device had undergone successful pilot testing.	Usual care – no intervention	757 caregivers were in the intervention group, 190 were in the control group.	9 months	Timeliness of immunisation rate (%) and reduction in dropout rate (%). **Categorised as timeliness.**	Pentavalent vaccine	
Schlumberger *et al*, 2015 [65].	Burkina Faso (West Africa)	Urban	RCT (2-arm) (Individual)	Automated ‘SMS-only’ appointment reminders	SMS appointment reminders sent before each of the eight immunisation sessions. Messages communicated that the caregiver was expected at the location of the appointment.	Usual care – no intervention	523 Mother/infant pairs (253 in the intervention group and 268 in control group).	5 months	Penta1, Penta2 and Penta3 coverage rate (%) and immunisation timeliness rate (%). **Both coverage and timeliness.**	Pentavalent vaccine	
Yunusa *et al*, 2022 [66].	Nigeria (West Africa)	Urban	Non-RCT (2-arm) (Clustered design)	‘SMS-only’ appointment reminders	Intervention group received SMS reminders 3days before their child’s next immunisation appointment, and on the day of appointment. Therefore, each mother/caregiver received 2x SMS for each of 3x Pentavalent doses.	Usual care – no intervention	541 Mother/infant pairs (271 in the intervention group and 270 in control group).	6 months	Penta1, Penta2 and Penta3 coverage rate (% and OR). **Categorised as coverage**.	Pentavalent vaccine	
Yunusa *et al*, 2024 [67].	Nigeria (West Africa)	Urban	RCT (2-arm) (Clustered design)	SMS and phone call reminders	Intervention group was sent 6x SMS and 3x phone call reminders at specific time intervals during study timeframe. 1st SMS was welcoming message, 2nd & 3rd were sent to participants in 4th & 5th week with their child’s appointment dates. 4th, 5th & 6th SMS reminders were sent 3x days before the scheduled date that their baby was due for immunisation (6, 10 & 14 weeks after birth).	Usual care – no intervention	554 Mother/infant pairs (277 in intervention group and 277 in control group, 18 were lost to follow up), so 536 Mother/infant pairs were analysed (275 in intervention group and 261 in control group).	14 weeks	Penta1, Penta 2 and Penta3 coverage (%) and Timeliness of immunisation (%). Both coverage and timeliness.	Pentavalent vaccine	Article in press

Note Penta1, Penta2 and Penta3 refers to 1st, 2nd and 3rd Pentavalent dose respectively. RR = Risk Ratio, OR = Odds Ratio, aOR = adjusted Odds Ratio, KES = Kenyan Shillings, BCG = Bacillus Calmette-Guérin vaccine, DTP = Diphtheria, Tetanus, and Pertussis vaccine, HCW = Health Care Worker, MCV = measles-containing vaccine, OPV = Oral Polio vaccine.

### Outcomes under investigation

The two key outcomes investigated in relation to vaccination uptake were vaccination coverage and timely receipt of doses. This review investigated mHealth/DH intervention effect on both these distinct but complementary outcomes to provide a more complete understanding of vaccination uptake. Including mHealth/DH intervention effect on coverage helped assess how well the target population was reached, while including intervention effect on timeliness provided insight into intervention effectiveness at ensuring vaccines were administered within the recommended time window, a critical factor for optimal immunity [41,42]. Seven studies included outcomes categorised as only relating to vaccination coverage [54–57,60,63,66]. Five studies measured both coverage and timeliness [58,59,61,65,67]. While only two studies only reported outcomes relating to timeliness [62,64].

### Study findings

Table 2 shows the key findings from the included studies. The two key parameters (vaccination coverage and timeliness), align with **Study Objectives 1** and **2**. As mentioned, each intervention from the 14 studies is presented individually. In total, 17 interventions related to coverage, and 12 interventions related to timeliness were analysed.

**Table 2:** The key findings from the 14 included studies relating to the 3 study objectives.

Author and year	Outcomes Measured (Coverage or Timeliness)	Results Relating to Study Objective 1 (Vaccination Coverage)	Results relating to Study Objective 2 (Vaccination Timeliness)	Additional Results relating to Study Objective 3	LogOR presented in Forest plots (Only 3^rd^ DTP/Pentavalent dose)	Overall Conclusions
Brown and Oluwatosin, 2017 [54].	Immunisation compliance rate (Considers the required number of doses at appropriate age and recommended intervals) (%). **Categorised as coverage.**	Immunisation compliance rate. **Intervention Group**: 79.2% **Control Group**: 46.4% (p < 0.001*).	N/A	98.2% of mothers/caregivers (n = 584) in the study population agreed to receive the immunisation reminder/recall phone calls. Therefore, was an acceptable intervention.	**Coverage:** LogOR 1.49 (95% CI 1.13–1.85) **Timeliness:** N/A	The results indicate that phone call reminders by health workers to mothers/caregivers can enhance immunisation compliance to the 3x dose schedule of DTP. * = statistical significance difference between the groups (p < 0.05).
Brown *et al*, 2016 [55].	Immunisation schedule completed or not completed (All essential immunisations including 3x doses of DTP/Pentavalent) (%, RR and OR). **Categorised as coverage.**	Immunisation schedule completion rate: **Group A (Phone call reminders)** = 98.6%. **Group C (Phone call reminders + Vaccinator training)** = 97.3%. **Group D (Control)** = 57.3%. RRs: **Group A vs Group D** = 1.72 (95% CI 1.5–1.98), **Group C vs Group D** = 1.70 (95% CI 1.47–1.95). ORs: **Group A **= 54.33 (95% CI 13.68–464.56, p < 0.0001*), **Group C **= 26.6 (95% CI 9.32–103.13, p < 0.0001*).	N/A	Vaccinator training alone (**Group B**) only marginally different to control group (RR 1.22, 95% CI 1.03–1.45). Group B compared to D, OR was 1.58 (95% CI 0.96–2.59) was not significant.	**Coverage:** **(A)** LogOR 3.99 (95% CI 2.56–5.43) **(C)** LogOR 3.28 (95% CI 2.24–4.33) **Timeliness:** N/A	The results show phone call reminders are associated with highest completion rate of the essential childhood immunisation schedule. Combining the intervention with training of the vaccinators was not superior to only using phone call reminders. This supports the implementation of phone call reminders in immunisation programmes to improve immunisation completion rates. * = statistical significance (p < 0.05).
Dissieka *et al*, 2019 [56].	Attendance at Penta1, Penta2 and Penta3 dose appointments (% and aOR). **Categorised as coverage.**	Immunisation appointment attendances. **Penta1: **Intervention group = 691 (86.6%), Control group = 607 (76.1%), (aOR = 2.85 (95% CI 1.85–4.37) (p < 0.001*). **Penta2: **Intervention = 646 (81%), Control = 537 (67.3%), aOR = 2.8 (95% CI 1.88–4.17) (p < 0.001*). **Penta3**: Intervention = 592 (74.2%), Control = 465 (58.3%), aOR = 2.68 (95% CI 1.84–3.91) (p < 0.001*).	N/A	In intervention Group 84.6% (n = 675) chose to receive a voice message reminder and 15.4% (n = 123) chose to receive SMS message reminders prior. Voice messages were preferred by 97.7% (n = 293) of mothers in rural areas. No significant difference between whether SMS or Voice message was used.	**Coverage:** LogOR 0.72 (95% CI 0.51–0.93) **Timeliness:** N/A	The results show that sending voice call or SMS appointment reminders increase attendance at each immunisation appointment. Majority of mothers chose voice messages in rural areas, suggesting that providing an option of SMS or voice call reminders would be most useful (particularly in more rural areas where literacy rates may be lower). * = statistical significance (p < 0.05).
Ekhaguere *et al*, 2019 [57].	Proportion of infants that received Penta1, Penta2 and Penta3 doses (% and RR). **Categorised as coverage.**	Pentavalent vaccine coverage. **Penta1**: Intervention = 285 (95%), Control = 289 (97%), RR was 0.98 (95% CI 0.95–1.02) p = 0.31. **Penta2**: Intervention = 276 (92%), Control = 278 (93%), RR was 0.99 (0.95–1.04) p = 0.76. **Penta3:** Intervention = 257 (86%), Control = 244 (81%), RR 1.05 (0.98–1.13) p = 0.15.	Pentavalent timeliness (receiving each within 1week of recommended time). **Penta1**: Intervention = 257 (86%), Control = 250 (83%), RR was 1.03 (95% CI 0.96–1.10, p = 0.43). **Penta2:** Intervention = 256 (85%), Control = 252 (84%), RR was 1.02 (0.95–1.09) p = 0.65. **Penta3:** Intervention = 253 (84%), Control = 233 (78%), RR was 1.09 (1.01–1.17, p = 0.04*).	Completing all immunisations by 12months of age was also recorded. Intervention = 171 (57%), Control = 140 (47%), RR was 1.22 (1.04–1.43, p = 0.01*).	**Coverage:** LogOR 0.32 (95% CI −0.12–0.75) **Timeliness:** LogOR 0.46 (95% CI 0.05–0.88)	No statistically significant difference was found between intervention group and control group for coverage of the 3x doses of Pentavalent vaccine (only significant difference in coverage was registered for MCV but this is not included in study). However, paired automated calls with SMS reminders significantly improved the proportion of infants that completed all routine immunisations by 12 months and significantly improved the timeliness of Penta3. * = statistical significance (p < 0.05).
Eze and Adeleye, 2015 [58].	DTP3 Coverage (%) and Timeliness of receipt (%). **Both coverage and timeliness.**	**DTP3** coverage = 69% for intervention group, 60.3% in control group.	DTP3 timeliness of receipt. Children were categorised into ‘early’ or ‘delayed’ (DTP3 is scheduled for 14th week, early = received DTP before 18weeks, missing appointment before 18weeks was categorised as delayed). **Early DTP3:** Intervention = 312 (69%), Control = vs 273 (60.3%), OR = 1.468 (95% CI 1.103–1.955).	N/A	**Coverage:** LogOR 0.38 (95% CI 0.11–0.66) **Timeliness:** LogOR 0.38 (95% CI 0.11–0.66)	Automated SMS and Voice message appointment reminders did increase the coverage of DTP3 vaccine; however, it is unclear whether this difference was statistically significant. The timeliness results showed that those in the intervention group were more likely to be ‘early’ than ‘delayed’ meaning the intervention did improve vaccination timeliness.
Gibson *et al*, 2017 [59].	Proportion of fully immunised children at 12months (included 3x Pentavalent doses plus 1x BCG, 3x OPV doses and 1x MCV) (% and RR) and timeliness of receipt (within 2weeks of recommended schedule) (% and RR). **Both coverage and timeliness.**	Full immunisation at 12months. **Group A** (‘SMS-only’) = 332 (86%), RR 1.04 (95% CI 0.97–1.12, p = 0.29). **Group B** (SMS + 75KES) = 383 (86%), RR 1.04 (95% CI 0.96–1.11, p = 0.33). **Group C** (SMS + 200KES) = 364 (90%), RR was 1.09 (95% CI 1.02–1.16, p = 0.0014*). **Group D** (Control) = 296 (82%). Pentavalent dose coverage. **Penta1:** All groups achieved 100%. **Penta2**: **Group A** = 383 (99%), RR 1.00 (95% CI 0.98–1.101, p = 0.77). **Group B **= 442 (99%), RR 1.00 (95% CI 0.99–1.02, p = 0.85). **Group C** = 404 (99.5%), RR 1.01 (95% CI 0.99–1.02, p = 0.42). **Group D** = 356 (99%). **Penta3: Group A** = 375 (97%), RR 0.98 (95% CI 0.96–1.01, p = 0.20). **Group B** = 439 (98%), RR 1.00 (95% CI 0.98–1.02, p = 0.82). **Group C** = 401 (99%), RR 1.00 (0.99–1.02, p = 0.58). **Group D** = 353 (98%).	Timeliness of Pentavalent doses (within 2weeks of recommended schedule). **Penta1**: **Group A** = 347 (89%), RR 0.98 (95% CI 0.94–1.03, p = 0.47). **Group B** = 412 (92%), RR 1.01 (95% CI 0.97–1.06, p = 0.55). **Group C** = 377 (93%), RR 1.02 (95% CI 0.98–1.06, p = 0.40). **Group D** = 328 (91%). **Penta2**: **Group A** = 320 (82%), RR 0.98 (95% CI 0.92–1.05, p = 0.54). **Group B** = 387 (87%), RR 1.03 (0.97–1.09, p = 0.31), **Group C** = 359 (88%), RR 1.05 (95% CI 0.99–1.11, p = 0.093). **Group D** = 303 (84%). **Penta3**: **Group A** = 288 (74%), RR 1.01 (95% CI 0.91–1.11, p = 0.90). **Group B** = 354 (79%), RR 1.07 (95% CI 0.98–1.17, p = 0.16). **Group C** = 337 (83%), RR 1.12 (95% CI 1.03–1.22, p = 0.0092*). **Group D** = 367 (74%).	Adding financial incentives did show significant improvement in timeliness of MCV (however not reported in this study).	**Coverage:** **(A)** LogOR −0.56 (95% CI −1.49–0.37) (**B)** LogOR 0.22 (95% CI −0.84–1.28) **(C)** LogOR 0.46 (95%CI −0.69–1.62) **Timeliness:** **(A)** LogOR 0.00 (95% CI −0.32–0.33) (**B**) LogOR 0.29 (95% CI −0.04–0.62) **(C)** LogOR 0.53 (95%CI 0.18–0.88)	The results show that combining SMS reminders plus cash incentives (‘SMS Plus’ intervention) were modestly effective at improving the proportion of children fully vaccinated by 12 months of age and SMS reminders (only incentivising with 200KES offered statistical significance), No significant difference was reported between the groups for Penta1, Penta2 or Penta3 coverage. These interventions were more effective at improving timeliness of Penta doses, with timeliness of receipt of Penta3 in Group C (SMS + 200KES) showing a significant difference, indicating larger cash amount is more favourable for this outcome. * = statistical significance (p < 0.05).
Haji *et al*, 2016 [60].	Penta2 and Penta3 coverage (%), and dropout rate (%). **Categorised as coverage.**	Pentavalent dose coverage. **Penta2**: Intervention = 365 (98%), Control = 340 (91%). **Penta3**: Intervention = 359 (96%), Control = 309 (83%).	Dropout Rate. **Intervention**: 365 (98%) received Penta2 and 359 (96%) received Penta3 dose (p = 0.4). **Control:** 340 (91%) received Penta2 and 309 (83%) received Penta3 (p < 0.001*). There was a significant increase in dropouts in control between Penta 2 and 3 but not in intervention.	N/A	**Coverage:** LogOR 1.73 (95% CI 1.11–2.34) **Timeliness:** N/A	The results show SMS reminders were effective in reducing dropouts for vaccinations in the selected Kenyan districts. The vaccination coverage was higher among those receiving SMS reminder than those receiving routine reminders. * = statistical significance (p < 0.05).
Ibraheem *et al*, 2021 [61].	Penta1, Penta2, Penta3 coverage and timeliness (%). Penta1, Penta2 and Penta3 timeliness (%) and likelihood of presentation for appointment (aOR). **Both coverage and timeliness.**	Pentavalent dose coverage. **Group A** (Phone call reminders) = 100% coverage for all 3x Pentavalent doses. **Group B** (‘SMS-only’ reminders) = 100% coverage for all 3x Pentavalent doses. **Group C** (‘SMS-Plus’ health education SMS) = 100% for Penta1 and Penta2. 134 (99.2%) for Penta3. **Group D** (Control), Penta1 = 131 (96.3%), Penta2 = 129 (94.9%), Penta3 = 127 (93.4%).	Timeliness of receipt of pentavalent doses (appropriate timing defined as 3days + /- scheduled appointment). **Penta1**: **Group A** = 132 (99.2%), **Group B** = 132 (97.1%). **Group C** = 133 (98.5%). **Group D** = 71 (54.1%), (Chi-square 175.643, p < 0.001*). **Penta2**: **Group A** = 124 (93.2%). **Group B** = 109 (80.1%). **Group C** = 117 (86.7%). **Group D** = 93 (72.1%), (Chi 22.935 p < 0.0001*). Only **Group A** had significant OR for Penta2 5.33 (95% CI 2.45–11.62, p < 0.001*). **Penta3**: **Group A** = 116 (87.2%). **Group B** = 95 (69.9%). **Group C** = 87 (64.9%). **Group D** = 85 (66.9%), (Chi 20.637 p < 0.001*). Only **Group A** had significant OR for Penta3 3.37 (95% CI 1.8–6.33, p < 0.001*).	Mother-infant pairs in all 3x intervention arms had significantly higher likelihood of presenting for subsequent immunisation visits than those in control group. **Group A**: aOR 35.4 (95% CI 4.83–259.56, p < 0.001*). **Group B**: aOR 36.2 (4.94–265.42, p < 0.001*). **Group C**: aOR 7.133 (2.77–18.38, p < 0.001*).	**Coverage:** **(A)** LogOR 2.99 (95% CI 0.14–5.84) (**B**) LogOR 3.01 (95% CI 0.16–5.87) **(C)** LogOR 2.25 (95% CI 0.17–4.33) **Timeliness:** **(A)** LogOR 1.41 (95% CI 0.79–2.03) (**B**) LogOR 0.33 (95% CI −0.18–0.83) **(C)** LogOR 0.08 (95%CI −0.41–0.58)	The results indicate that phone call reminders are associated with the highest appropriateness of presentation timing for vaccinations. All 3x mHealth intervention groups were more likely to present for subsequent appointments, therefore these interventions might be more useful for immunisations requiring multiple doses. * = statistical significance (p < 0.05).
Kawakatsu *et al*, 2020 [62].	Return rate for subsequent immunisation appointment (% and aOR). **Categorised as timeliness.**	Unadjusted return rate for subsequent immunisation appointment. **Intervention**: 67% (95% CI 66%−68%). **Control:** 62% (95% CI 61% − 64%) (p < 0.001*). aOR 1.17 (95% CI 1.05–1.31, p < 0.05*) for return visits.	Number of return visits (subsequent appointments) on original appointment date. **Intervention:** 2038 (41.7%). **Control:** 1228 (35.7%) (p < 0.001*).	N/A	**Coverage:** N/A **Timeliness:** LogOR 0.25 (95% CI 0.16–0.34)	The results show that sending SMS text reminder is more likely to increase return visit rate. Mothers/caregivers are also more likely to show up for return visits and stick to original scheduled appointment dates, indicating the benefits for schedule adherence. * = statistical significance (p < 0.05).
Oladepo *et al*, 2020 [63].	Completion of all immunisations (%) and coverage of Penta1, Penta2 and Penta3 (%). **Categorised as coverage.**	All immunisation completion rate. **Intervention:** 76% **Control:** 73.3%, (p = 0.00*). Pentavalent Dose Coverage: **Penta1:** Intervention = 1082 (86.9%), Control = 938 (80.7%), (p = 0.00*). **Penta2:** Intervention = 1076 (87.5%), Control = 856 (74.8%), (p = 0.00*). **Penta3:** Intervention = 1035 (85%), Control = 799 (70.6%), (p = 0.00*).	N/A	N/A	**Coverage:** LogOR 0.86 (95% CI 0.65–1.06) **Timeliness:** N/A	The results show that there is a significant association between the intervention and completion of the 3x pentavalent vaccine doses. This can be attributable to the reminder messages intervention. * = statistically significant association (Chi-square).
Sampson *et al*, 2023 [64].	Timeliness of immunisation rate (%) and reduction in dropout rate (%). **Categorised as timeliness.**	N/A	Immunisation timeliness rate. **Intervention:** Baseline timeliness = 35% (289/827), Endline timeliness = 69% (522/757). Intervention effective at improving immunisation timeliness. **Control:** Baseline timeliness = 64% (104/162), Endline timeliness = 67% (127/190). No statistical tests were conducted.	60% reduction in dropout rate was recorded in intervention group between baseline and endline, and a 21% reduction in dropout was registered in the control group.	**Coverage:** N/A **Timeliness:** LogOR 0.10 (95% CI 0.24–0.44)	Immunisation alert wristband intervention was shown to be effective at improving immunisation timeliness and reducing dropouts during immunisation schedule. It is certainly an interesting intervention that shows promise for mothers/caregivers with lower literacy and digital literacy.
Schlumberger *et al*, 2015 [65].	Penta1, Penta2 and Penta3 coverage rate (%) and immunisation timeliness rate (%). **Both coverage and timeliness.**	Pentavalent dose coverage. **Penta1**: Intervention = 187 (73.3%), Control = 150 (55.7%), (p < 0.001*). **Penta2:** Intervention = 182 (71.3%), Control = 144 (53.6%), (p < 0.001*). **Penta3:** Intervention = 165 (60.3%), Control = 126 (42.3%), (p < 0.001*).	Pentavalent dose timeliness. **Penta1:** Intervention = 113 (60.4%), Control = 73 (48.7%), (p = 0.03*). **Penta2**: Intervention = 81 (60.4%), Control = 49 (48.7%), (p = 0.02*). **Penta3**: Intervention = 45 (29.2%), Control = 29 (25.4%), (p = 0.49).	N/A	**Coverage:** LogOR 0.75 (95% CI 0.4–1.10) **Timeliness:** LogOR 0.58 (95% CI 0.08–1.08)	The results show that the SMS intervention was effective at increasing coverage of all 3x Penta doses in Burkina Faso context in comparison to control. The intervention led to significant improvement in increasing Penta1 and Penta2 timeliness, however, no significant difference was associated with Penta3 timeliness. * = statistical significance (p < 0.05).
Yunusa *et al*, 2022 [66].	Penta1, Penta2 and Penta3 coverage rate (% and OR). **Categorised as coverage**.	Pentavalent dose coverage. **Penta1**: Intervention = 210 (77.4%), Control = 194 (71.9%), OR 1.349 (95% CI 0.914–1.991, p = 0.132). **Penta2**: Intervention = 185 (68.3%), Control = 132 (48.9%), OR 2.249 (85% CI 1.585–3.191, p < 0.0001*). **Penta3**: Intervention = 163 (60.1%), Control = 117 (43.3%), OR 1.974 (95% CI 1.402–2.779, p < 0.0001*). Completion of all 3x Penta doses: Intervention = 161 (59.4%), Control = 92 (34.1%), OR 2.832 (95% CI 1.997–4.016, p < 0.0001*).	N/A	Recommended that researchers use the local languages in sending the reminders to parents to enhance understanding.	**Coverage:** LogOR 0.68 (95% CI 0.34–1.02) **Timeliness:** N/A	The results show that SMS combine with phone call immunisation reminders are effective in improving the completeness of all 3x pentavalent vaccine immunisation in the studied population, especially over time with the 2nd and 3rd doses. * = statistical significance (p < 0.05).
Yunusa *et al*, 2024 [67].	Penta1, Penta 2 and Penta3 coverage (%) and Timeliness of immunisation (%). **Both coverage and timeliness.**	Pentavalent dose coverage. **Penta1**: Intervention = 196 (71.3%), Control = 133 (50.9%), (p < 0.001*). **Penta2**: Intervention = 175 (63.6%), Control = 75 (28.7%), (p < 0.001*). **Penta3**: Intervention = 169 (61.5%), Control = 44 (16.9%), (p < 0.001*).	Pentavalent dose timeliness (receipt of immunisation within a month from the due date of recommended schedule). **Penta1**: Intervention = 170 (61.8%), Control = 70 (26.8%), (p < 0.001*). **Penta2**: Intervention = 149 (54.2%), Control = 37 (14.2%), (p < 0.001*). **Penta3**: Intervention = 145 (52.7%), Control = 20 (7.7%), (p < 0.001).	N/A	**Coverage:** LogOR 2.06 (95% CI 1.66–2.47) **Timeliness:** LogOR 2.60 (95% CI 2.08–3.11)	The results show that combined SMS and phone call reminders are effective in improving the completeness and timeliness of all 3x pentavalent doses for childhood immunisation in Nigeria. Article is in press. * = statistical significance (p < 0.05).

Note Penta1, Penta2 and Penta3 refers to 1st, 2nd and 3rd Pentavalent dose respectively, and DTP3 refers to third DTP dose. 95% CI = 95% Confidence Intervals, RR = Risk Ratio, OR = Odds Ratio, aOR = adjusted Odds Ratio, KES = Kenyan Shillings, BCG = Bacillus Calmette-Guérin vaccine, DTP = Diphtheria, Tetanus, and Pertussis vaccine, MCV = Measles-containing vaccine, OPV = Oral polio vaccine.

### Risk of bias assessments

**Fig 3:**
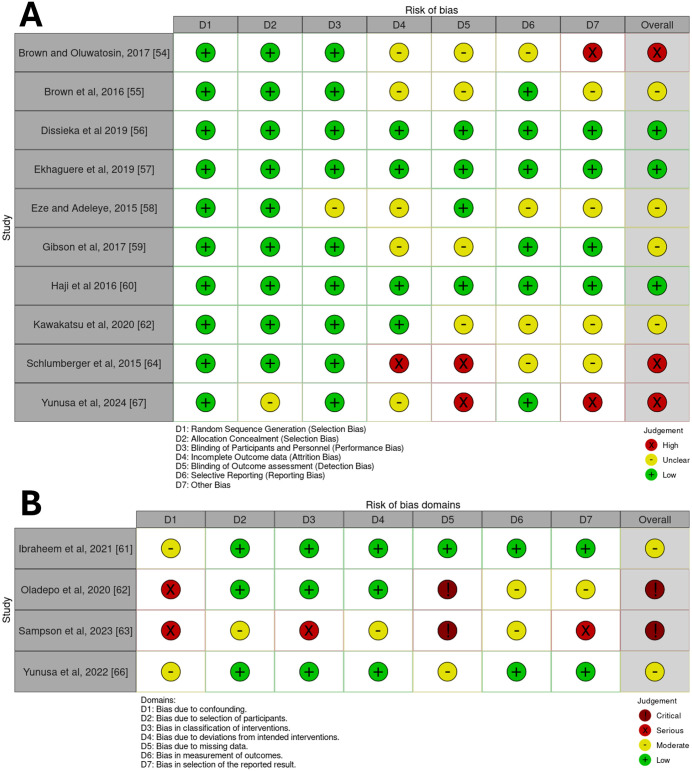
The risk of bias assessments of the included studies. **(A)** Risk of bias assessment of the RCTs using RoB2 [47], note that D7 ‘Other biases’ column refers to assessments of other important biases not assessed by RoB2, including assessing study population baseline imbalances, study design appropriateness, potential early stoppage of trials and potential conflicts of interest or funding biases **(B)** Risk of bias assessments of the non-RCTs using ROBINS-I [48].

Fig 3 shows the results of the RoB2 and ROBINS-I risk of bias assessments. Three of the included RCTs were considered as ‘Low risk’ [56,57,60]. All three of these studies were categorised as reporting outcomes relating to vaccination coverage, suggesting the findings relating to this outcome may have greater internal validity. Four were scored as having ‘Some concerns’, reporting on a mix of coverage, timeliness or both outcomes [55,58,59,61]. Three were considered at ‘High risk’ [54,65,67]. Amongst the included non-RCTs, none were considered ‘Low risk’, two were scored as having ‘Moderate risk’ [61,66], and two were considered as having ‘Critical risk’ of bias [63,64]. This spread of risk of bias ratings highlights substantial variability in study quality, particularly among non-RCTs. The concentration of lower risk of bias studies reporting on vaccination coverage suggests more reliable conclusions can be made for this outcome. In contrast, caution is warranted when interpreting findings from studies assessed as having high or critical risk of bias (reported on both outcomes of interest). The presence of multiple studies with ‘some concerns’ or higher further emphasises the need for more rigorous, high-quality research to strengthen the evidence base.

### Study Objective 1 Findings – mHealth/DH intervention effect on vaccination coverage

**Fig 4:**
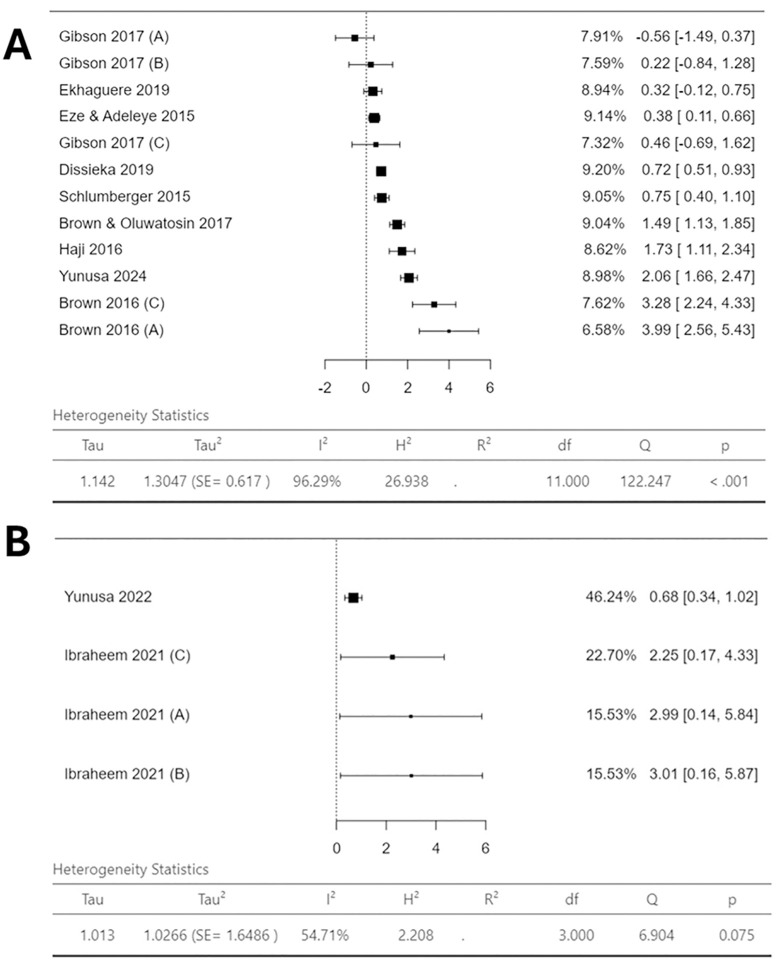
Forest plots showing logOR for mHealth/DH intervention effectiveness for increasing vaccination coverage of DTP/Pentavalent3. **(A)** logOR point estimates for the included RCTs **(B)** logOR point estimates for included non-RCTs*. % weighting of each study is shown which correlates to heterogeneity statistics along with 95% confidence intervals of the point estimates. As each intervention was analysed individually ‘Gibson 2017 **(A)**’ relates to Group A’s intervention from Gibson *et al* (2017) [59]. *Note Oladepo *et al* (2020) [63] has been excluded as an outlier as it had a weighting of 72.98% which resulted in masked heterogeneity (I^2^ = 0%). This large influence in the plot diminishes variability between studies.

Fig 4 provides a visual representation of the mHealth/DH interventions effectiveness in improving vaccination coverage of DTP/Pentavalent3 by study design.

Fig 4A shows the outcomes from the included RCTs. Extremely high heterogeneity was reported (I^2^ = 96.29%). Eleven interventions from the RCTs demonstrated a positive association between intervention and vaccination coverage. Only ‘Gibson 2017 (A)’, reported a negative outcome (−0.56 logOR, 95% CI −1.49 to 0.37) [59]. However, the confidence interval crosses the null (logOR = 0), indicating potential for a positive association. There are two outliers (which likely contribute to the elevated I^2^ statistic) displaying notably high effect estimates: ‘Brown 2016 (A)’ (3.99 logOR, 95% CI 2.56 to 5.43) and ‘Brown 2016 (C)’ (3.28 logOR 95% CI 2.24 to 4.33), both are phone call reminder interventions [55]. The remaining point estimates show a more homogeneous range, from 0.22 logOR (95% CI −0.84 to 1.28) to 2.06 logOR (95% CI 1.66 to 2.47). Overall, these findings indicate a likely positive association between mHealth/DH interventions and vaccination coverage.

Fig 4B shows associations between mHealth/DH interventions and vaccination coverage amongst the included non-RCTs. All four interventions presented here show positive logOR point estimates, suggesting these interventions were likely associated with increased vaccination coverage. Oladepo *et al* (2020) reported 0.86 logOR (95% CI 0.65–1.06) but was not included in the forest plot as its large weighting (due to sample size) masks heterogeneity (I^2^ = 0%), its overwhelming influence diminishes variability between studies [63]. With this excluded Fig 4B still displays moderate-high heterogeneity (I^2^ = 54.71%), thus grounds for narrative synthesis still stands.

### Study Objective 2 Findings – mHealth/DH intervention effect on vaccination timeliness

**Fig 5:**
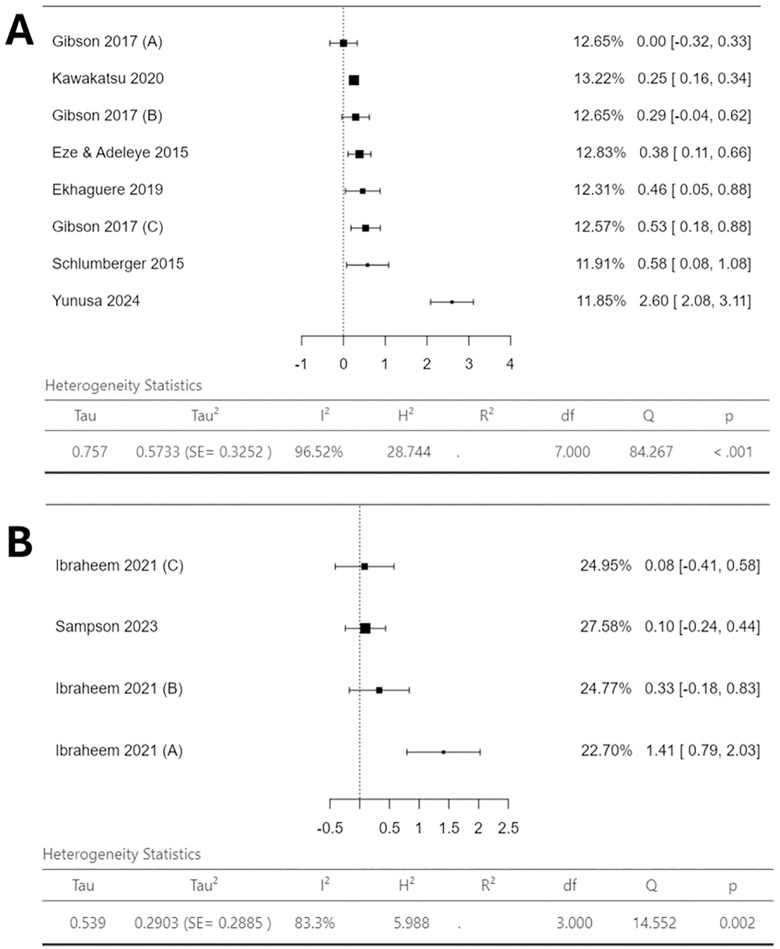
Forest plots showing logOR for mHealth/DH intervention effectiveness for improving timeliness of administration of DTP/Pentavalent3. **(A)** logOR point estimates for the included RCTs **(B)** logOR point estimates for included non-RCTs. % weighting of each study is shown which correlates to heterogeneity statistics along with 95% confidence intervals of the point estimates.

Fig 5 provides visual representation of the mHealth/DH intervention effect on timeliness of administration of DTP/Pentavalent3 by study design.

Fig 5A illustrates the interventions effect on timeliness from the included RCTs. Again, an extremely high I^2^ is reported (96.52%). These interventions showed fairly homogenous results, ranging from 0.00 logOR (95% CI −0.32 to 0.33) to 0.58 logOR (95% CI 0.08 to 1.08), indicating a positive association between mHealth/DH intervention and DTP/Pentavalent3 timeliness. Yunusa *et al* (2024) was a sole outlier (SMS and Phone call reminder intervention), reporting enhanced point estimate of 2.60 logOR (95% CI 2.08 to 3.11) [67].

Fig 5B shows the interventions investigated amongst the non-RCTs effect on timeliness. A high I^2^ is also reported (83.3%). Group B and C of Ibraheem *et al* (2021) and Sampson et al (2023), show fairly homogenous point estimates ranging from 0.08 logOR (95% CI −0.41 to 0.58) to 0.33 logOR (95% CI −0.18 to 0.83) [61,64]. The only outlier was Group A of Ibraheem *et al* (2021), showing 1.41 logOR (95% CI 0.79 to 2.03) [61].

### Study Objective 3 Findings – Additional results relevant to mHealth/DH intervention implementation

Two studies provided useful additional information [56,66]. Dissieka *et al* (2019) reported that when given a choice between receiving SMS or voice message appointment reminders, 675 (84.6%) of mothers/caregivers chose to receive voice message reminders, whereas only 123 (15.4%) chose to receive SMS messages, and voice message reminders were preferred by 97.7% in rural areas [56]. Additionally, Yunusa *et al* (2022) recommended sending SMS messages in local languages, and to not assume English is understood, an important consideration for future intervention development [66].

### GRADE certainty of evidence assessment

The GRADE assessment was conducted separately around each of the intervention-type subgroups and the two primary outcome groups (vaccination coverage and vaccination timeliness). Table 3 shows the GRADE summary of findings table. The full explanations for GRADE scoring are shown in S5 File. In the vaccination coverage outcome group, the findings from ‘SMS-only’ and ‘SMS reminders and/or Voice messages or Phone calls’ subgroups were scored as having ‘Moderate’ certainty. The ‘Phone call-only’ subgroup scored ‘Low’ certainty, whilst the ‘SMS-Plus’ subgroup’s findings were considered ‘Very low’ certainty. For the vaccination timeliness findings, the ‘SMS-only’ and ‘SMS-Plus’ intervention subgroups’ findings were considered as having ‘Moderate’ certainty, whereas the ‘SMS reminders and/or Voice messages or Phone calls’, ‘Phone call-only’ and ‘Electronic Immunisation Alert Wristband’ all scored ‘Very low’ certainty.

**Table 3:** GRADE Summary of findings table showing certainty of evidence for each outcome and intervention-type pairings.

Outcome	Intervention Type Subgroup	Study Design (RCT or Non-RCT)	Risk of Bias	Inconsistency	Indirectness	Imprecision	Publication Bias	Effect Estimate Range (LogOR (95% CI))	Certainty of Evidence (GRADE)
Vaccination Coverage	SMS-only	Both, RCT (n=4) & Non-RCT (n=2)	Serious Concerns	No Serious Concerns	No Serious Concerns	No Serious Concerns	No Serious Concerns	-0.56 LogOR (95% CI -1.49-0.37) to 3.01 LogOR (95% CI 0.16-5.87)	**Moderate **⊗⊗⊗
	SMS-Plus	Both, RCT (n=2) & Non-RCT (n=2)	Very Serious Concerns	Serious Concerns	Serious Concerns	Very Serious Concerns	No Serious Concerns	0.22 LogOR (95% CI -0.84-1.28) to 2.25 LogOR (95% CI 0.17-4.33)	**Very Low **⊗
	SMS reminders and/or Voice messages or Phone calls	RCT (n=3)	Serious Concerns	No Serious Concerns	No Serious Concerns	No Serious Concerns	No Serious Concerns	0.32 LogOR (95% CI -0.12-0.75) to 2.06 LogOR (95% CI 1.66-2.47)	**Moderate **⊗⊗⊗
	Phone call-only	Both, RCT (n=3) & Non-RCT (n=1)	Serious Concerns	No Serious Concerns	No Serious Concerns	Some Concerns	Serious Concerns	1.49 LogOR (95% CI 1.13-1.85) to 3.99 LogOR (95% CI 2.56-5.43)	**Low **⊗⊗
Vaccination Timeliness	SMS-only	Both, RCT (n=3) & Non-RCT (n=1)	No Serious Concerns	No Serious Concerns	No Serious Concerns	Serious Concerns	No Serious Concerns	0.00 LogOR (95% CI -0.32-0.33) to 0.38 LogOR (95% CI 0.11-0.66)	**Moderate **⊗⊗⊗
	SMS-Plus	Both, RCT (n=2) & Non-RCT (n=1)	No Serious Concerns	No Serious Concerns	Serious Concerns	No Serious Concerns	No Serious Concerns	0.08 LogOR (95% CI -0.41-0.58) to 0.53 LogOR (95% CI 0.18-0.88)	**Moderate **⊗⊗⊗
	SMS reminders and/or Voice messages or Phone calls	RCT (n=2)	Serious Concerns	Serious Concerns	No Serious Concerns	Some Concerns	Serious Concerns	0.46 LogOR (95% CI 0.05-0.88) to 2.60 LogOR (95% CI 2.08-3.11)	**Very Low **⊗
	Phone call-only	Non-RCT (n=1)	No Serious Concerns	No Serious Concerns	No Serious Concerns	Serious Concerns	Serious Concerns	1.41 LogOR (95% CI 0.79-2.03)	**Very Low **⊗
	Electronic Wristband	RCT (n=1)	Very Serious Concerns	No Serious Concerns	No Serious Concerns	Serious Concerns	Serious Concerns	0.10 LogOR (95% CI -0.24-0.44)	**Very Low **⊗

*See S5 File for full explanations on scoring domains.

### Narrative synthesis

For the narrative synthesis the studies have been grouped by mHealth/DH intervention-type. The description and which studies included in each subgroup is shown in S4 File.

### Study Objective 1 Narrative Synthesis – mHealth/DH intervention effect on vaccination coverage

Four mHealth/DH intervention-type subgroups reported outcomes categorised as vaccination coverage (‘SMS-only’ appointment reminders, ‘SMS-Plus’, ‘SMS reminders and/or Voice messages or Phone calls’, and ‘Phone call-only’ reminders).

(i) *‘SMS-only’ appointment reminders (including automated and non-automated)*

Five ‘SMS-only’ interventions showed a positive association with vaccination coverage [58,60,61,65,66]. Group A in Gibson *et al* (2017) reported a negative logOR (−0.56 (95% CI −1.49 to 0.37)) [59], however, as the confidence interval crosses the null, a positive association might be possible. Contrastingly, Group B of Ibraheem *et al* (2021) demonstrated a much greater point estimate of 3.01 logOR (95% CI 0.16 to 5.87) [61]. However, the broad confidence interval range suggests imprecision in the point estimate, which may limit the reliability of this finding. The remaining four studies all showed a positive association between ‘SMS-only’ reminders and vaccination coverage, ranging from 0.38 logOR (95% CI 0.11 to 0.66) to 1.71 logOR (95% CI 1.11 to 2.34) [58,60,65,66]. The intervention-outcome group pairing scored moderate certainty of evidence; therefore, we have moderate confidence in these findings and recognise that further research may lead to alternative conclusions.

Overall, the results suggest that implementing ‘SMS-only’ appointment reminders may have a modest positive effect on vaccination coverage in this context. For ‘SMS-only’ interventions to be effective, mothers/caregivers must receive the SMS reminder [68]. If a spouse or relative’s phone is used, then this opens the possibility of reminders being missed, however, it was unclear in included studies whether SMS reminders were received. As the SMS messages must be read and understood by recipients to be effective, the modest results exhibited could be explained because of lower literacy rates amongst recipients, particularly in rural or underserved communities where literacy rates might be lower [69,70]. These barriers (shared phone ownership and low literacy) may limit SMS-only intervention effectiveness, therefore must be considered when implementing SMS-based reminders to improve childhood vaccination uptake.

(ii) *‘SMS-Plus’ (SMS appointment reminders plus cash incentives or health education SMS messages)*

The ‘SMS-Plus’ interventions show great variation in logOR, likely due to the diversity of intervention enhancements. Oladepo *et al* (2020) reported the most precise point estimate, 0.86 logOR (95% CI 0.65 to 1.06), however, due to potential limitations identified in risk of bias assessment, we acknowledge this finding’s reliability may be reduced [63]. Group B and C of Gibson *et al* (2017), which combined SMS reminders with cash incentives, reported 0.22 logOR (95% CI −0.84 to 1.28) and 0.46 logOR (95% CI −0.69 to 1.62), respectively [59]. Confidence intervals in both crossed the null, reflecting great uncertainty in point estimate precision. Group C of Ibraheem *et al* (2021), showed 2.25 logOR (95% CI 0.17 to 4.33), with a wide confidence interval range, again indicating extreme uncertainty in point estimate precision [61]. Overall, the certainty of evidence for this intervention-outcome pairing was very low, which limits the strength of conclusions regarding the effectiveness of ‘SMS-Plus’ interventions for increasing vaccination coverage.

The findings suggest that ‘SMS-Plus’ interventions may offer some benefit for increasing vaccination coverage, but current evidence is limited by imprecision and variability in intervention design. An interesting observation was that health education intervention reported by Oladepo et al (2020), showed greater effect in comparison to cash incentivisation interventions for vaccination coverage [61,63]. As ‘SMS-Plus’ interventions are also SMS-based, they also require SMS receipt and recipient understanding of SMS contents to be effective, this could explain the similar outcomes to ‘SMS-only’ interventions. As interventions were ‘enhanced’ through cash incentivisation or health educational messaging, stronger associations between interventions and outcomes were expected, however, were not found. Given the additional resource requirements, further research is needed to assess the contextual appropriateness and cost-effectiveness of these enhanced interventions in improving childhood immunisation uptake across SSA.

(iii) *‘SMS reminders and/or Voice messages or Phone calls’*

All three studies in this subgroup showed positive associations between intervention and vaccination coverage with varying degrees of precision [56,57,67]. Yunusa *et al* (2024) reported the strongest association, logOR of 2.06 (95% CI 0.17 to 4.33), however, this finding’s validity may be influenced by the risk of bias assessment and should be interpreted accordingly [67]. Ekhaguere *et al*’s (2019) intervention (combining SMS and voice messages), reported a positive 0.32 logOR (95% CI −0.12 to 0.75), however, as the confidence interval range crosses the null there is uncertainty around the estimate’s precision [57]. Dissieka *et al* (2019) carried the most weight when considering risk of bias assessment, reporting 0.72 logOR (95% CI 0.51 to 0.93) [56]. The point estimate suggests that providing mothers/caregivers with a choice of SMS or voice messages is positively associated with increased vaccination coverage. For this intervention-outcome group, the certainty of evidence was again moderate, indicating sufficient confidence that these interventions will positively affect vaccination coverage.

These results suggest combining SMS reminders with voice-based communication (voice messages or phone calls), or allowing recipients to choose between the two, may be a more effective strategy for improving vaccination coverage. The positive associations observed may reflect the ability of voice-based reminders to overcome literacy-related barriers. Additionally, offering caregivers a choice could increase engagement by promoting a sense of autonomy and empowerment in relation to their child’s health [71]. Given the moderate certainty of evidence and promising findings, these interventions merit serious consideration in future immunisation programmes. However, attention should also be paid to feasibility and cost-effectiveness in different contexts given their more resource-intensive nature.

(iv) *‘Phone call-only’ reminders*

All three interventions involving phone call reminders show great positive associations for vaccination coverage [54,55,61]. All point estimates were >1.00. Brown & Oluwatosin (2017) provided the most precise estimate in the subgroup, with logOR of 1.49 (95% CI, 1.13 to 1.85) [54]. Group A in Ibraheem *et al* (2021), reported 2.99 logOR (95% CI, 0.14 to 5.84), however the wide confidence interval range indicates substantial uncertainty in this point estimate’s precision, raising concerns around its reliability [61]. Group A of Brown *et al* (2016), reported highest logOR of 3.99 (95% CI, 2.56 to 5.43) [55]. Although the GRADE assessment rated this intervention-outcome group as having low certainty of evidence, the point estimates were consistently greater than those of other intervention-types, suggesting that phone call reminder interventions may offer the greatest potential for increasing vaccination coverage. While these findings support the hypothesis that interventions incorporating voice-based components may be more effective and appear well-suited to contexts where literacy or access to SMS technology is limited, the low certainty of evidence limits their immediate applicability for informing public health policy and practice. These findings reinforce the value of direct, human-to-human contact in promoting health behaviours. Further high-quality studies are needed to confirm effectiveness and explore implementation challenges, including workforce capacity and cost considerations.

### Study Objective 2 Narrative Synthesis – mHealth/DH intervention effect on vaccination timeliness

Five mHealth/DH intervention-type subgroups reported outcomes categorised as vaccination timeliness (‘SMS-only’ appointment reminders, ‘SMS-Plus’, ‘SMS reminders and/or Voice messages or Phone calls’, ‘Phone call-only’ reminders, and ‘Electronic immunisation alert wristband’).

(i) *‘SMS-only’ appointment reminders (including automated and non-automated)*

Three ‘SMS-only’ interventions showed positive associations between intervention and vaccination timeliness [58,61,62]. Group A of Gibson *et al* (2017), showed no effect, 0.00 logOR (95% CI −0.32 to 0.33), however, the wide confidence interval range, indicates uncertainty around true point estimate [59]. The remaining point estimates were fairly homogeneous showing slight positive associations, with Kawakatsu *et al* (2020) reporting 0.25 logOR (95% CI 0.16 to 0.34) [62], and Group B of Ibraheem *et al* (2021) reporting 0.33 logOR (95% CI −0.18 to 0.83) [61]. Although Ibraheem *et al* (2021) reported a positive association here, the wide confidence interval range crosses the null, indicates potential for no effect [61]. Eze and Adeleye (2015) also reported a slight positive association of 0.38 (95% CI 0.11 to 0.66) [58]. A moderate certainty of evidence was found for this intervention-outcome pairing. Similar to the vaccination coverage findings, ‘SMS-only’ interventions appear likely to be more effective than no intervention but may only lead to modest improvements in vaccination timeliness. The same contextual factors, such as literacy and phone access, may explain the limited effect sizes.

(ii) *‘SMS-Plus’ (SMS appointment plus cash incentives or health education SMS)*

All three interventions showed positive associations between intervention and vaccination timeliness [59,61]. Group C of Ibraheem et al (2021), which received health education SMS, reported only a slight positive association of 0.08 logOR (95% CI −0.41 to 0.58) [61], with the wide confidence interval range suggesting the possibility of no effect. Groups B and C of Gibson et al (2017) combined SMS appointment reminders with cash incentives of 75KES and 200KES respectively [59]: Group B reported 0.29 logOR (95% CI −0.04 to 0.62), however, again the confidence interval crossed the null, whereas Group C demonstrated a stronger positive association of 0.53 logOR (95% CI 0.18 to 0.88). These findings were assessed as having moderate certainty, indicating satisfactory confidence. While combining SMS appointment reminders with larger cash incentives may enhance vaccination timeliness, more research, particularly cost-effectiveness analyses, is required to support decisions on enhancing interventions for increasing vaccination timeliness.

(iii) *‘SMS reminders and/or Voice messages or Phone calls’*

Only two interventions in this subgroup reported outcomes categorised as timeliness [57,67]. Yunusa et al (2024) reported a strong association, logOR of 2.60 (95% CI 2.08 to 3.11) [67], while Ekhaguere et al’s (2019) intervention (combining SMS and voice messages), reported a smaller logOR of 0.46 (95% CI, 0.05 to 0.88) [57]. This intervention-outcome group was assessed as having very low certainty of evidence, indicating the true effect may be substantially different from the findings reported here.

Nonetheless, the results suggest that combining SMS reminders with a voice-based component may have a positive effect on vaccination timeliness, by potentially overcoming some of the limitations associated with ‘read-only’ SMS interventions. However, caution is advised given the limited number of studies and the low certainty.

(iv) *‘Phone call-only’ reminders*

Group A of Ibraheem et al (2021) was the sole ‘Phone call-only’ reminder intervention reporting on timeliness with a logOR of 1.41 (95% CI, 0.79 to 2.03) [61]. It’s relatively narrow confidence intervals and satisfactory risk of bias, suggests a likely positive effect on vaccination timeliness. While this aligns with study findings, the very low certainty of evidence rating means that uncertainty remains, and further high-quality research is needed to strengthen the evidence base.

(v) *‘Electronic immunisation alert wristband’*

Only one study implemented the wearable electronic immunisation alert wristband, and reported a slight positive association, 0.10 logOR (95% CI, −0.24 to 0.44) [64]. The confidence interval crosses the null, indicating potential for no effect. Again, this intervention received a very low certainty of evidence rating.

## Discussion

### Overview

This systematic review and subsequent narrative synthesis investigated which mHealth/DH interventions are most effective at increasing vaccination uptake (of DTP/Pentavalent vaccine) in the 19 SSA countries that were due to roll out malaria vaccine in 2024. Our aim was to identify which mHealth/DH intervention types were most effective at improving childhood vaccination coverage and timeliness in contexts where new vaccine rollouts are planned, and to provide guidance on the development and use of mHealth/DH interventions in future SSA vaccination programmes. Most studies were conducted in West Africa (ten from Nigeria [54,55,57,58,61–64,66,67], one from Burkina Faso [65], and one from Cote D’Ivoire [56]), making the findings more generalisable to that setting. The remaining two studies were conducted in Kenya, East Africa [59,60]. The included mHealth/DH interventions were all related to communication technology and immunisation appointment reminders. The intervention-types investigated included: ‘SMS-only’ appointment reminders, ‘SMS-Plus’ appointment reminders, ‘SMS reminders and/or Voice messages or Phone calls’, ‘Phone call-only’ appointment reminders, and a wearable 'Electronic immunisation alert wristband'. Extremely high heterogeneity was observed across studies, which is common in mHealth/DH intervention research and has been noted in similar reviews [33,36]. Thus, a narrative synthesis was conducted by grouping mHealth/DH intervention-type to assess their effectiveness for improving vaccination coverage and timeliness.

A positive association between intervention and improvement in vaccination coverage was reported for 16 interventions (Fig 3), and 11 interventions showed a positive association between intervention and vaccination timeliness of DTP/Pentavalent3 administration (Fig 4). This consistency in findings across the included studies, enhances the evidence’s reliability. However, since almost all reported interventions showed a positive association, concerns around publication bias must be acknowledged. Conducting a more rigorous grey literature search and searching of trial registries may have reduced potential publication bias and increase this review’s external validity [72]; however, this was outside the scope of this review. Despite this limitation, the review provides robust evidence for informing policy and practice on mHealth/DH intervention implementation in SSA childhood immunisation programmes.

### SMS-based interventions (‘SMS-only’ and ‘SMS-Plus’)

When considering intervention-type subgroups, the findings suggest that voice-based interventions (phone call or voice message reminders) are likely to be more effective than SMS-based interventions. Only slight positive associations were observed for both vaccination coverage and timeliness in ‘SMS-only’ appointment reminder interventions, with moderate certainty of evidence. This suggests that sending out ‘SMS-only’ appointment reminders is more favourable than no intervention, however, other types of mHealth/DH intervention may be more effective at improving vaccination coverage and timeliness. The stronger ‘SMS-only’ positive associations were reported in urban areas, indicating potentially greater generalisability to these settings.

‘SMS-Plus’ interventions also showed positive associations, however, stronger associations in comparison to ‘SMS-only’ interventions were not found despite ‘SMS-Plus’ interventions being enhanced. The certainty of evidence for the effect of ‘SMS-Plus’ interventions on vaccination coverage was very low, highlighting uncertainty in the findings. Whereas the intervention–outcome group for ‘SMS-Plus’ and vaccination timeliness was assessed as having moderate certainty of evidence, indicating much greater reliability in findings for that outcome.

Interestingly, although some ‘SMS-Plus’ interventions offered mothers/caregivers cash incentives for attending immunisation appointments, it was the SMS health educational interventions that demonstrated stronger associations with improved vaccination coverage. When considering SMS reminders combined with cash incentives, offering a larger incentive (200KES = ~1.2GBP), appeared to have greater effect on both vaccination coverage and timeliness compared to the lower amount (75KES = ~0.45GBP) [59]. However, according to the GRADE assessment, the evidence supporting these interventions for improving vaccination timeliness was more reliable. In the wider literature, providing mothers with 2USD when their child received timely pentavalent vaccine doses, was found to increase the proportion of individuals receiving timely pentavalent doses [73]. However, this study utilised a small study population, lowering its validity. In contrast, another systematic review, specifically investigating financial incentives for increasing coverage of child health interventions, found limited effect on immunisation coverage when financial incentives were offered, indicating this may not be a useful intervention [74]. Interestingly, an RCT in Ghana found that offering small financial incentives to health care workers improved vaccination coverage and timeliness of BCG and OPV [75], suggesting that incentivising those administering vaccinations might be more effective.

A major limitation of both ‘SMS-only’ and ‘SMS-Plus’ interventions is the possibility that target recipients do not receive the SMS reminders. The ability to log SMS delivery was beyond the scope of the included studies, and assumptions were made on target population receiving SMS messages. This presents an opportunity for future research investigating mHealth SMS intervention delivery success rate in SSA context. Additionally, for SMS-based interventions to be successful, recipients must read and understand the respective messages, highlighting another potential limitation. Low literacy amongst mothers/caregivers could result in them not understanding the contents of SMS reminders, severely limiting their effectiveness. ‘SMS-Plus’ interventions involving sending educational messages around vaccination may be particularly susceptible to this limitation. This could be particularly prominent in rural communities where literacy rates might be lower. The intervention showing least effect in the ‘SMS-only’ subgroup was conducted in a rural area which supports this hypothesis, therefore is an important consideration [59]. The potential limitation around literacy was also reported in a SMS-based data collection study amongst midwives in Liberia, it concluded SMS interventions must be targeted towards those with higher literacy [76]. Although, a certain literacy level is required for these interventions to be effective, new mothers/caregivers should be encouraged to seek help if illiterate, and strategies to complement SMS-based interventions developed to address this barrier. A study in rural Kenya highlighted the importance of keeping SMS messages simple and easy to understand [77]. Investigating the association between participant educational attainment and mother/caregiver literacy and SMS immunisation appointment reminder effectiveness, perhaps through an observational case-control study, would provide valuable insight into this potential limitation.

Despite these limitations, it could be argued that implementing basic SMS-based interventions increases accessibility and may have wider reach. Although SMS-based interventions have fewer capabilities (e.g., extremely limited multi-media content delivery and tracking capabilities), SMS-compatible devices are cheaper and are supported on basic ‘second generation’ 2G cellular connectivity networks which are much more widely available in rural areas [78]. Therefore, when considering connectivity and affordability barriers, implementing basic interventions may be more equitable.

The findings in this review, along with supporting evidence from the wider literature, suggest that ‘SMS-only’ interventions are likely to offer some benefit for improving vaccination outcomes, particularly where more advanced infrastructure or resources are lacking. Given their potential low cost and ease of implementation, such interventions may be a useful entry point in settings preparing for new vaccine rollouts [78]. The results also indicate that enhancing SMS interventions with educational content (‘SMS-Plus’) may improve vaccination coverage more effectively than adding cash incentives, while the inclusion of cash incentives may be more effective for improving timeliness, potentially by creating a sense of urgency around appointment attendance. However, larger cost-effectiveness analyses are needed to determine whether the additional resources required for enhanced interventions deliver sufficient value, particularly in resource-constrained settings.

### Voice-based interventions (‘SMS reminders and/or Voice messages or Phone calls’ and ‘Phone call-only’)

In contrast to SMS-only interventions, those incorporating a voice-based component, such as phone calls or voice messages, demonstrated stronger associations with improvements in both vaccination coverage and timeliness.

The intervention showing the strongest association combined SMS and phone call reminders, suggesting that implementing combination would likely be effective for increasing vaccination coverage and timeliness [67]. The certainty of evidence for this intervention-type subgroup was assessed as moderate for vaccination coverage, and very low for vaccination timeliness, indicating that recommendations are more reliable for the vaccination coverage context. When considering risk of bias, Dissieka et al’s (2019) finding was considered the most reliable in the subgroup [56]. Their RCT was conducted in rural Cote D’Ivoire and offered participants the choice of receiving SMS or voice message reminders. Interestingly, most participants chose to receive voice messages, demonstrating that when given the choice, voice messages were the preferred intervention. This finding supports the hypothesis that effectiveness of SMS-based interventions is potentially limited in rural populations with lower literacy. Although Cote D’Ivoire does have relatively high literacy rates in comparison to other SSA nations, female literacy is lower [79]. As mothers/caregivers are the main target of these intervetions, this could explain the finding and further highlights the importance of considering target demographic literacy, particularly in rural settings, when developing mHealth/DH interventions. It is acknowledged that only this one study offered participants a choice of intervention, thus limiting its generalisability. However, it does highlight another research gap and need for further investigation into providing choices of intervention in the immunisation programme context.

Using voice messages has been successful in other contexts in SSA, for example a Nigerian RCT demonstrated that women receiving voice messages were more likely to attend antenatal care appointments [80]. Furthermore, they were implemented in rural Senegal to improve infant and child feeding practices [81]. This certainly highlights the potential for voice-message interventions; however, we recognise that combining SMS interventions with voice-based components may be more resource-intensive. Again, further cost-effectiveness study would help clarify its viability.

Overall, the findings demonstrate that these interventions are likely to be effective for increasing vaccination coverage in routine childhood immunisation programmes, and that offering participants a choice of intervention (SMS or voice message) should be considered during intervention development.

Generally, the positive associations reported in the ‘Phone call-only’ subgroup were stronger in comparison to the other subgroups. This further supports the hypothesis that interventions with voice-based components are more effective. This could be due to recipients responding better to more engaging and personal interventions [82]. Implementing phone calls also creates the opportunity for recipients to potentially seek clarification on queries relating to vaccination and may allow reinforcement messaging about importance of vaccination and adhering to outlined vaccination schedules by vaccinators. Furthermore, using phone calls would overcome the previously outlined limitations of SMS-based reminders, with verbal information more likely to be understood. A Cochrane review investigating various patient reminder interventions, mirrored this review’s findings reporting that phone call reminders were the most effective intervention [83]. However, they did raise the concern around phone call reminders being more costly than other methods. While interventions incorporating phone call reminders appear to be most effective in increasing vaccination coverage and timeliness and show promise, further study is required to increase the findings’ validity.

Despite the presented evidence showing this intervention is likely to be effective, context-specific cost-effectiveness comparisons between SMS and phone call-based reminders should be conducted to inform future vaccination programmes. Another systematic review reported that phone call reminders were effective, however, two of their included studies reported that costs per SMS reminder were considerably lower than phone calls, again highlighting this intervention’s potentially costly nature [84]. With expansion of AI and voice technology, the cost-barriers of these interventions may be reduced and should be strongly considered for future immunisation programmes following cost-effectiveness analysis [85].

### Other interventions

The final intervention subgroup was the ‘Electronic immunisation alert wristband’. Although an interesting intervention, only a very slight positive association was shown. Its imprecision and very low certainty of evidence mean recommendations cannot be made supporting this intervention. A similar intervention in Pakistan also showed limited effect [86].

### Implications for mHealth/DH intervention development

Based on the review’s findings, implementing mHealth/DH interventions appears to hold promise for improving childhood vaccination coverage and timeliness in SSA context. Interventions that combine SMS appointment reminders with voice-based components, such as phone calls or voice messages, emerged as potentially more effective than SMS-only approaches, particularly in settings with lower literacy. While these approaches may be more resource-intensive, they may offer a more equitable and engaging solution, especially in rural or lower-literacy populations. Therefore, future mHealth/DH immunisation reminder interventions should, where feasible, consider incorporating these elements.

### Transferability of findings to the malaria vaccine context

Although this review synthesised evidence from interventions aimed at increasing uptake of the DTP/Pentavalent vaccine, the included countries were selected based on their involvement in the planned RTS,S/AS01 and R21/Matrix-M malaria vaccine rollouts in SSA. The introduction of the first malaria vaccines represents a momentous milestone in public and global health, with the potential to significantly reduce malaria-related morbidity and mortality among children under five [1,87,88]. As such, these findings may provide useful insights to inform the design of mHealth/DH interventions in that context and support improved uptake and timeliness of the new malaria vaccines. Despite the DTP/Pentavalent vaccines and the RTS,S/AS01 and R21/Matrix-M malaria vaccines both being multi-dose vaccines delivered via intramuscular injection, we must acknowledge fundamental differences in these vaccine types and immunisation age profiles. For example, as the DTP/Pentavalent vaccines are delivered at 6, 10, and 14 weeks of age, mHealth/DH interventions to increase uptake of these are targeting increased uptake of vaccine coverage and timeliness in a child’s early infancy [19,20]. Contrastingly, despite requiring multiple doses, the malaria vaccines are administered to children later, from around five months of age, through a three-dose primary schedule, followed by a booster dose after 12–18 months, meaning recipients are much older and care givers may have differing health seeking priorities depending on their baby’s age [87–89]. Thus, we recognise that the findings generated in this review may be more applicable to immunisation programmes for children in early infancy, such as the primary schedules of the EPI.

This review focused specifically on interventions targeting DTP/Pentavalent vaccines because these are the most widely implemented multi-dose vaccines across the EPI in SSA, providing a consistent and comparable evidence base across countries [21,90]. Furthermore, DTP/Pentavalent3 coverage is often used as a key metric to measure the performance of a country’s routine immunisation programme, with high DTP/Pentavalent3 coverage indicating that the health system is effectively reaching infants with the full multi-dose series [21,90]. We acknowledge, however, that the contextual factors influencing uptake of DTP/Pentavalent vaccines, typically administered in early infancy, may differ from those affecting multi-dose vaccines delivered later in childhood, such as the Measles-Containing Vaccine (MCV) or Measles, Mumps and Rubella (MMR) vaccine [21,91]. Interventions targeting these later-age vaccines may therefore offer insights that are more directly comparable to the malaria vaccines, which are administered at older ages. SMS text message reminder systems have been shown in the literature to be effective for MCV. Mekonnen et al. (2019) reported that SMS reminders significantly improved both coverage and timeliness for MCV, with timely coverage increasing from 79.3% to 91.5% (p < 0.001) in the intervention group compared with usual care [92]. Furthermore, an RCT in Kenya assessed the impact of SMS reminders, with and without small unconditional monetary incentives, on timely uptake of the first MCV dose. The study found that both SMS-only and SMS-plus-cash incentive interventions increased timely vaccination coverage compared with the control group, though only the SMS and cash incentive arm achieved a statistically significant effect [93]. This suggests that ‘SMS-Plus’ approaches, where SMS reminders are complemented by an additional component such as a monetary incentive, may be particularly effective for vaccines administered at older ages, such as the malaria vaccines. This represents an important route for future investigation.

While it is plausible that interventions effective for improving DTP/Pentavalent vaccine coverage and timeliness could have similar effects for malaria vaccines, we acknowledge that disease characteristics and differences in immunisation age schedules mean that further context-specific research is needed before drawing definitive conclusions. Nevertheless, the findings from this review provide useful, evidence-based insights to inform the design and implementation of mHealth/DH interventions in future multi-dose vaccination programmes across SSA.

### Findings in comparison to other systematic reviews

As previously mentioned, several similar systematic reviews were identified during scoping searches. Gilano *et al* (2024) investigated the effect of mHealth on all essential childhood immunisations. Despite reporting extremely high heterogeneity, they conducted a meta-analysis and reported an OR of 2.21 for Penta3 dose [36]. Our narrative findings mirror this and further support use of mHealth/DH interventions to increase vaccination coverage. Eze *et al* (2021) solely focused on SMS appointment reminders in LMICs in general and like our review focused on DTP/Pentavalent vaccine [33]. Again, despite reporting high heterogeneity, a meta-analysis found that implementing SMS reminders significantly improved childhood immunisation coverage (RR = 1.16, 95% CI 1.10 to 1.21) and timeliness (RR = 1.21, 95% CI 1.12 to 1.30) [33]. Our systematic review did find similar conclusions that mHealth/DH interventions of all types including SMS appointment reminders, were likely to improve vaccination coverage and timeliness. The authors recognised phone call reminders would be advantageous for reaching populations with limited or no education, and future studies should explore a combined intervention for optimising immunisation outcomes. This complements the ideas generated by in review, further increasing the evidence-base.

### Geographic generalisability of results in SSA

This review aimed to synthesise evidence on mHealth/DH interventions for increasing uptake of DTP/Pentavalent vaccines in the 19 SSA countries targeted to roll out malaria vaccines in 2024. However, studies from only four of these countries were included, meaning 15 were not represented, which limits the generalisability of the findings.

Most included studies were conducted in Nigeria, which likely reflects the country’s large population, and active mHealth/DH research landscape [94,95]. Therefore, the findings may be particularly applicable to the Nigerian context, where previous research has indicated that mHealth/DH interventions are generally acceptable [96–98]. However, challenges such as low mobile phone ownership and literacy rates, particularly in rural Nigerian communities show the need to consider these context-specific factors during intervention design to ensure equitable implementation [99].

Although 15 SSA countries were not represented in our review, there is evidence showing mHealth/DH interventions are being implemented in these countries. A comparative review on mHealth and eHealth use in SSA included studies from 17 SSA countries [100]. It reported Uganda, South Africa, Madagascar and Kenya as the SSA countries with the most mHealth/DH studies. Aboye et al (2023), also found Uganda to be most represented (along with Kenya and Nigeria) [5]. Whilst South Africa and Madagascar were out with this review’s scope, it is questionable why Uganda was not represented here. There is evidence of mHealth/DH use in Uganda in the context of vaccination, for example using DHIS2 [101]. Additionally, one of the excluded studies in this review was an RCT protocol on SMS to reduce vaccination dropouts in Uganda, showing research is happening there [102]. mHealth/DH interventions have also been used in more of the 15 SSA countries not represented here such as Ghana, Sierra Leone, and South Sudan [75,103,104]. This suggests that implementing a mHealth/DH intervention in childhood immunisation programmes in these countries would likely be effective, however, it is acknowledged that country-specific communication preferences should be explored.

Although this review focused on the stated 19 SSA countries, it is important to acknowledge that valuable mHealth/DH research is also being conducted in other countries across SSA. For example, countries such as Ethiopia, which were not within the review’s inclusion scope, are emerging as key contributors to this research area [92]. For example, an RCT in Northwestern Ethiopia, found that mHealth text message reminders improved both Penta3 coverage (95.8% vs 86.9%, p < 0.001) and timeliness of administration (88.7% vs 69.2%, p < 0.001) compared with no intervention, demonstrating this mHealth intervention’s potential effectiveness in Ethiopian context [92]. Future reviews may benefit from expanding geographic inclusion to capture insights from a broader range of SSA contexts.

### Impact and important considerations

It is well established that essential childhood vaccinations contribute hugely to public health preventing millions of deaths annually, and finding strategies to increase their uptake and coverage, whilst ensuring their timely administration is essential to maximise their protective benefits. Although many of the mHealth/DH interventions in this review showed only a small effect, when scaled to population level they could have great positive impact. As full rollout of the malaria vaccines began in 2024, there is huge potential to alleviate some of the malaria-attributed childhood mortality and morbidity, and the presented evidence in this review suggests that mHealth/DH interventions could support health systems and immunisation programmes in SSA to increase vaccine reach. However, country-specific studies on intervention acceptability and feasibility in both urban and rural settings, must be conducted to assess their effectiveness in specific contexts. Additionally, the findings highlight the need for considerations around target population literacy and technology accessibility, particularly in relation to implementing SMS-based interventions. Implementing voice-based interventions may overcome some of these barriers, however, questions around costs may limit feasibility in more resource limited settings. It must be acknowledged that accessibility and affordability of mobile devices remain significant barriers in LMICs, with many people lacking mobile phone access or sufficient digital literacy [105–107]. High mobile data connectivity costs in LMICs are also potentially prohibitive, which would limit these interventions [32,107]. These challenges are arguably more prevalent in low-income rural populations, potentially excluding vulnerable groups from beneficial health interventions [105,107]. Despite the clear advantages of mHealth/DH interventions, care must be taken during intervention development not to exclude populations and avoid exacerbating inequity and growing the ‘digital divide’ [32].

### Limitations

A key limitation of this review is the limited transferability of findings from interventions targeting DTP/Pentavalent vaccination to other immunisation programmes, particularly the malaria vaccine context. Although both are childhood vaccines, DTP/Pentavalent vaccines are administered in early infancy, whereas the malaria vaccines have a different disease profile and are delivered later in infancy (typically from around five months of age). Therefore, while the findings may offer useful insights, they should be interpreted with caution when applied to the malaria vaccine rollout. Overall, the review provides valuable evidence-based insights for the use of mHealth/DH interventions in SSA immunisation programmes, however, several other key limitations must be acknowledged. For example, only published literature was included, highlighting potential publication bias. A more thorough search of the unpublished material or clinical trial registries would have reduced this limitation but was not possible due to resource constraints. Additionally, it is recognised that in a narrative synthesis, although guided by quantitative findings, there is possibility for subjectivity in selection of findings which could have led to reporting bias, however, the included certainty of evidence assessment considers the potential impact of reporting and publication bias on the confidence in presented results. In terms of study findings, it is recognised that by limiting the study context to only 19 SSA countries, the search breadth and inclusion criteria was limited and excluded countries such as South Africa, Ethiopia and Tanzania where mHealth/DH research is occurring [100]. Another limitation is generalisability of findings, with Nigeria being disproportionately represented here. Although Kenya, Burkina Faso and Cote D’Ivoire were represented, the overall finding’s transferability to these countries is also limited due to study low numbers. Although the included studies were from a mix of urban and rural settings, it must be noted that different countries and settings likely have different preferences and cultural factors for communications. For example, it was shown that Kenyan farmers preferred phone calls over SMS [82].

## Conclusion

This systematic review demonstrates that mHealth/DH interventions can improve childhood vaccination coverage and timeliness in SSA. Interventions combining SMS reminders with voice-based components show particular promise as they may overcome some of the outlined limitations associated with SMS-only interventions.

While the available evidence highlights the potential of mHealth/DH interventions to improve vaccination uptake, these insights should be interpreted in light of the underlying certainty of evidence and the review’s outlined limitations in generalisability and transferability. Nonetheless, the review’s findings offer valuable evidence-based insights to guide development and implementation of mHealth/DH interventions within SSA immunisation programmes. As countries across SSA begin to introduce the malaria vaccines into their routine childhood immunisation programmes, these insights may be especially relevant for informing future intervention design; however, further context-specific research is required to draw more definitive conclusions.

## Supporting information

S1 File PRISMA checklist. (DOCX)

S2 File PICOS, inclusion/exclusion criteria. (DOCX)

S3 File Search strategies. (DOCX)

S4 File Included studies and description of intervention-type Subgroups. (DOCX)

S5 File GRADE certainty of evidence assessment explanations. (DOCX)

S6 File Raw quantitative data for Forest Plot. (DOCX)

## References

[1] WHO. WHO - Nearly 10 000 children vaccinated as malaria vaccine rollout in Africa expands. https://www.afro.who.int/news/nearly-10-000-children-vaccinated-malaria-vaccine-rollout-africa-expands. Accessed 2024 August.

[2] Dumit EM, Novillo-Ortiz D, Contreras M, Velandia M, Danovaro-Holliday MC. The use of eHealth with immunizations: An overview of systematic reviews. Vaccine. 2018;36(52):7923–8. doi: 10.1016/j.vaccine.2018.06.076 29983255

[3] Mc Kenna P, Broadfield LA, Willems A, Masyn S, Pattery T, Draghia-Akli R. Digital health technology used in emergency large-scale vaccination campaigns in low- and middle-income countries: a narrative review for improved pandemic preparedness. Expert Rev Vaccines. 2023;22(1):243–55. doi: 10.1080/14760584.2023.2184091 36814067

[4] WHO. mHealth - Use of appropriate digital technologies for public health. 2018. https://apps.who.int/gb/ebwha/pdf_files/WHA71/A71_20-en.pdf?ua=1#:~:text=Digital%20health%20and%20specifically%20mHealth,of%20acute%20and%20chronic%20diseases

[5] Aboye GT, Vande Walle M, Simegn GL, Aerts J-M. mHealth in sub-Saharan Africa and Europe: A systematic review comparing the use and availability of mHealth approaches in sub-Saharan Africa and Europe. Digit Health. 2023;9. doi: 10.1177/20552076231180972 37377558 PMC10291558

[6] Osei E, Mashamba-Thompson TP. Mobile health applications for disease screening and treatment support in low-and middle-income countries: A narrative review. Heliyon. 2021;7(3):e06639. doi: 10.1016/j.heliyon.2021.e06639 33869857 PMC8035664

[7] Aranda-Jan CB, Mohutsiwa-Dibe N, Loukanova S. Systematic review on what works, what does not work and why of implementation of mobile health (mHealth) projects in Africa. BMC Public Health. 2014;14:188. doi: 10.1186/1471-2458-14-188 24555733 PMC3942265

[8] Marcolino MS, Oliveira JAQ, D’Agostino M, Ribeiro AL, Alkmim MBM, Novillo-Ortiz D. The Impact of mHealth Interventions: Systematic Review of Systematic Reviews. JMIR Mhealth Uhealth. 2018;6(1):e23. doi: 10.2196/mhealth.8873 29343463 PMC5792697

[9] WHO. WHO guideline recommendations on digital interventions for health system strengthening. 1. Geneva: World Health Organization. 2019. https://www.ncbi.nlm.nih.gov/books/NBK541905/31162915

[10] Chan J. Exploring digital health care: eHealth, mHealth, and librarian opportunities. J Med Libr Assoc. 2021;109(3):376–81. doi: 10.5195/jmla.2021.1180 34629965 PMC8485950

[11] Ibeneme S, Karamagi H, Muneene D, Goswami K, Chisaka N, Okeibunor J. Strengthening Health Systems Using Innovative Digital Health Technologies in Africa. Front Digit Health. 2022;4:854339. doi: 10.3389/fdgth.2022.854339 35434700 PMC9008130

[12] Oliver-Williams C, Brown E, Devereux S, Fairhead C, Holeman I. Using Mobile Phones to Improve Vaccination Uptake in 21 Low- and Middle-Income Countries: Systematic Review. JMIR Mhealth Uhealth. 2017;5(10):e148. doi: 10.2196/mhealth.7792 28978495 PMC5647459

[13] Olu O, Muneene D, Bataringaya JE, Nahimana M-R, Ba H, Turgeon Y, et al. How Can Digital Health Technologies Contribute to Sustainable Attainment of Universal Health Coverage in Africa? A Perspective. Front Public Health. 2019;7:341. doi: 10.3389/fpubh.2019.00341 31803706 PMC6873775

[14] Dolan SB, Wittenauer R, Shearer JC, Njoroge A, Onyango P, Owiso G, et al. Integration of a Digital Health Intervention Into Immunization Clinic Workflows in Kenya: Qualitative, Realist Evaluation of Technology Usability. JMIR Form Res. 2023;7:e39775. doi: 10.2196/39775 36917157 PMC10131705

[15] Jepson R, McAteer J, Williams AJ, Doi L, Buelo A. Developing public health interventions: a step-by-step guide. SAGE Publications Ltd STM. 2022.

[16] WHO. Vaccines and immunizations fact sheet. https://www.who.int/health-topics/vaccines-and-immunization#tab=tab_1. Accessed 2024 March.

[17] WHO. Essential Programme on Immunization. https://www.who.int/teams/immunization-vaccines-and-biologicals/essential-programme-on-immunization. 2024. Accessed 2025 August.

[18] Sreedhar S, Antony A, Poulose N. Study on the effectiveness and impact of pentavalent vaccination program in India and other south Asian countries. Hum Vaccin Immunother. 2014;10(7):2062–5. doi: 10.4161/hv.28785 25424816 PMC4186033

[19] Dimitrova A, Carrasco-Escobar G, Richardson R, Benmarhnia T. Essential childhood immunization in 43 low- and middle-income countries: Analysis of spatial trends and socioeconomic inequalities in vaccine coverage. PLoS Med. 2023;20(1):e1004166. doi: 10.1371/journal.pmed.1004166 36649359 PMC9888726

[20] Pollard AJ, Edwards KM, Fritzell B. Rationalizing childhood immunization programs: The variation in schedules and use of combination vaccines. New generation vaccines. CRC Press. 2016:458–70.

[21] Oyo-Ita A, Oduwole O, Arikpo D, Effa EE, Esu EB, Balakrishna Y, et al. Interventions for improving coverage of childhood immunisation in low- and middle-income countries. Cochrane Database Syst Rev. 2023;12(12):CD008145. doi: 10.1002/14651858.CD008145.pub4 38054505 PMC10698843

[22] Akmatov MK, Mikolajczyk RT. Timeliness of childhood vaccinations in 31 low and middle-income countries. J Epidemiol Community Health. 2012;66(7):e14. 10.1136/jech.2010.124651 21551179 21551179

[23] Mihigo R, Okeibunor J, Anya B, Mkanda P, Zawaira F. Challenges of immunization in the African Region. Pan Afr Med J. 2017;27(Suppl 3):12. doi: 10.11604/pamj.supp.2017.27.3.12127 29296147 PMC5745929

[24] Ortiz JR, Robertson J, Hsu J-S, Yu SL, Driscoll AJ, Williams SR, et al. The potential effects of deploying SARS-Cov-2 vaccines on cold storage capacity and immunization workload in countries of the WHO African Region. Vaccine. 2021;39(15):2165–76. doi: 10.1016/j.vaccine.2021.02.037 33744049 PMC7894202

[25] Wallace AS, Willis F, Nwaze E, Dieng B, Sipilanyambe N, Daniels D, et al. Vaccine wastage in Nigeria: An assessment of wastage rates and related vaccinator knowledge, attitudes and practices. Vaccine. 2017;35(48 Pt B):6751–8. doi: 10.1016/j.vaccine.2017.09.082 29066189 PMC5771486

[26] Sato R, Thompson A, Sani I, Metiboba L, Giwa A, Femi-Ojo O, et al. Effect of Vaccine Direct Delivery (VDD) on vaccine stockouts and number of vaccinations: Case study from Bauchi State, Nigeria. Vaccine. 2021;39(9):1445–51. doi: 10.1016/j.vaccine.2021.01.037 33541796

[27] Matos CC de SA, Gonçalves BA, Couto MT. Vaccine hesitancy in the global south: Towards a critical perspective on global health. Glob Public Health. 2022;17(6):1087–98. doi: 10.1080/17441692.2021.1912138 33843459

[28] Muathe EC, Kamau M, Rajula E. Exploring Strategies to Improve Adherence to Immunization Schedule: A Study among Children Attending Maternal and Child Health Clinic at Kenyatta National Hospital, Nairobi, Kenya. Int J Pediatr. 2020;2020:4730205. doi: 10.1155/2020/4730205 32849883 PMC7439158

[29] Hampshire K, Mwase-Vuma T, Alemu K, Abane A, Munthali A, Awoke T, et al. Informal mhealth at scale in Africa: Opportunities and challenges. World Dev. 2021;140:105257. doi: 10.1016/j.worlddev.2020.105257 33814676 PMC7903241

[30] Betjeman TJ, Soghoian SE, Foran MP. mHealth in Sub-Saharan Africa. Int J Telemed Appl. 2013;2013:482324. doi: 10.1155/2013/482324 24369460 PMC3867872

[31] GSMA. The Mobile Economy Sub-Saharan Africa 2023. 2023. https://www.gsma.com/solutions-and-impact/connectivity-for-good/mobile-economy/wp-content/uploads/2024/05/ME-SSA-2023.pdf

[32] Hansen C, Ringel M, Evans L, Evans J. Leave no community behind: the digital divide. Digital Respiratory Healthcare. European Respiratory Society. 2023. doi: 10.1183/2312508x.10001123

[33] Eze P, Lawani LO, Acharya Y. Short message service (SMS) reminders for childhood immunisation in low-income and middle-income countries: a systematic review and meta-analysis. BMJ Glob Health. 2021;6(7):e005035. doi: 10.1136/bmjgh-2021-005035 34290051 PMC8296799

[34] Metiboba L, Katuka A, Adam T, Giwa Z, Fagge R, Haidar N. Analyzing the effectiveness of digital technology in vaccine supply management in Kano State, Nigeria. 2023.

[35] Mvundura M, Di Giorgio L, Lymo D, Mwansa FD, Ngwegwe B, Werner L. The costs of developing, deploying and maintaining electronic immunisation registries in Tanzania and Zambia. BMJ Glob Health. 2019;4(6):e001904. doi: 10.1136/bmjgh-2019-001904 31803511 PMC6882552

[36] Gilano G, Sako S, Molla B, Dekker A, Fijten R. The effect of mHealth on childhood vaccination in Africa: A systematic review and meta-analysis. PLoS One. 2024;19(2):e0294442. doi: 10.1371/journal.pone.0294442 38381753 PMC10880990

[37] Ekhaguere O, Oluwafemi RO, Mendonca EA. 3 A mobile health-supported bundle to improve routine childhood vaccine completion rate in Nigeria. J Clin Trans Sci. 2024;8(s1):1–1. doi: 10.1017/cts.2024.27

[38] Higgins JPT, Thomas J, Chandler J, Cumpston M, Li T, Page MJ, Welch VA (editors). Cochrane Handbook for Systematic Reviews of Interventions version 6.5 (updated August 2024). Cochrane, 2024. Available from www.training.cochrane.org/handbook

[39] Page MJ, McKenzie JE, Bossuyt PM, Boutron I, Hoffmann TC, Mulrow CD, et al. The PRISMA 2020 statement: an updated guideline for reporting systematic reviews. BMJ. 2021;372:n71. doi: 10.1136/bmj.n71 33782057 PMC8005924

[40] Methley AM, Campbell S, Chew-Graham C, McNally R, Cheraghi-Sohi S. PICO, PICOS and SPIDER: a comparison study of specificity and sensitivity in three search tools for qualitative systematic reviews. BMC Health Serv Res. 2014;14:579. doi: 10.1186/s12913-014-0579-0 25413154 PMC4310146

[41] Dolan SB, Carnahan E, Shearer JC, Beylerian EN, Thompson J, Gilbert SS, et al. Redefining vaccination coverage and timeliness measures using electronic immunization registry data in low- and middle-income countries. Vaccine. 2019;37(13):1859–67. doi: 10.1016/j.vaccine.2019.02.017 30808566 PMC6420680

[42] Walton S, Cortina-Borja M, Dezateux C, Griffiths LJ, Tingay K, Akbari A, et al. Measuring the timeliness of childhood vaccinations: Using cohort data and routine health records to evaluate quality of immunisation services. Vaccine. 2017;35(51):7166–73. doi: 10.1016/j.vaccine.2017.10.085 29132992 PMC5720480

[43] OpenHIE.org. http://www.ohie.org. Accessed 2024 September.

[44] OpenMRS.org. www.openmrs.org. 2024. Accessed 2024 September.

[45] WHO. WHO digital health and innovation team publications. www.who.int/teams/digital-health-and-innovation. 2024. Accessed 2024 September.

[46] Frank J, Jepson R, Williams AJ. Seeing the forest for the trees—finding and using the evidence. Disease Prevention. Oxford University Press. 2016:36–54. doi: 10.1093/med/9780198725862.003.0004

[47] Sterne JAC, Savović J, Page MJ, Elbers RG, Blencowe NS, Boutron I, et al. RoB 2: a revised tool for assessing risk of bias in randomised trials. BMJ. 2019;366:l4898. doi: 10.1136/bmj.l4898 31462531

[48] Sterne JA, Hernán MA, Reeves BC, Savović J, Berkman ND, Viswanathan M, et al. ROBINS-I: a tool for assessing risk of bias in non-randomised studies of interventions. BMJ. 2016;355:i4919. doi: 10.1136/bmj.i4919 27733354 PMC5062054

[49] Schünemann HJ, Higgins JP, Vist GE, Glasziou P, Akl EA, Skoetz N, et al. Completing ‘Summary of findings’ tables and grading the certainty of the evidence. Cochrane Handbook for Systematic Reviews of Interventions. Wiley. 2019. 375–402. doi: 10.1002/9781119536604.ch14

[50] Prasad M. Introduction to the GRADE tool for rating certainty in evidence and recommendations. Clinical Epidemiology and Global Health. 2024;25:101484. doi: 10.1016/j.cegh.2023.101484

[51] Campbell M, McKenzie JE, Sowden A, Katikireddi SV, Brennan SE, Ellis S, et al. Synthesis without meta-analysis (SWiM) in systematic reviews: reporting guideline. BMJ. 2020;368:l6890. doi: 10.1136/bmj.l6890 31948937 PMC7190266

[52] Cruz-Retamozo X, Prado-Ghezzi D, Pereyra-Elías R. Forest Plots: Linear or Logarithmic Scale?. J Adolesc Health. 2017;61(5):664–5. doi: 10.1016/j.jadohealth.2017.07.025 29061236

[53] Hu D, Wang C, O’Connor AM. A method of back-calculating the log odds ratio and standard error of the log odds ratio from the reported group-level risk of disease. PLoS One. 2020;15(3):e0222690. doi: 10.1371/journal.pone.0222690 32126072 PMC7053742

[54] Brown VB, Oluwatosin OA. Feasibility of implementing a cellphone-based reminder/recall strategy to improve childhood routine immunization in a low-resource setting: a descriptive report. BMC Health Serv Res. 2017;17(Suppl 2):703. doi: 10.1186/s12913-017-2639-8 29219093 PMC5773899

[55] Brown VB, Oluwatosin OA, Akinyemi JO, Adeyemo AA. Effects of Community Health Nurse-Led Intervention on Childhood Routine Immunization Completion in Primary Health Care Centers in Ibadan, Nigeria. J Community Health. 2016;41(2):265–73. doi: 10.1007/s10900-015-0092-3 26395786

[56] Dissieka R, Soohoo M, Janmohamed A, Doledec D. Providing mothers with mobile phone message reminders increases childhood immunization and vitamin A supplementation coverage in Côte d’Ivoire: A randomized controlled trial. J Public Health Afr. 2019;10(1):1032. doi: 10.4081/jphia.2019.1032 31285815 PMC6589636

[57] Ekhaguere OA, Oluwafemi RO, Badejoko B, Oyeneyin LO, Butali A, Lowenthal ED, et al. Automated phone call and text reminders for childhood immunisations (PRIMM): a randomised controlled trial in Nigeria. BMJ Glob Health. 2019;4(2):e001232. doi: 10.1136/bmjgh-2018-001232 31139442 PMC6509606

[58] Eze GU, Adeleye OO. Enhancing Routine Immunization Performance using Innovative Technology in an Urban Area of Nigeria. West Afr J Med. 2015;34(1):3–10. 26902809

[59] Gibson DG, Ochieng B, Kagucia EW, Were J, Hayford K, Moulton LH, et al. Mobile phone-delivered reminders and incentives to improve childhood immunisation coverage and timeliness in Kenya (M-SIMU): a cluster randomised controlled trial. Lancet Glob Health. 2017;5(4):e428–38. doi: 10.1016/S2214-109X(17)30072-4 28288747 PMC5348605

[60] Haji A, Lowther S, Ngan’ga Z, Gura Z, Tabu C, Sandhu H, et al. Reducing routine vaccination dropout rates: evaluating two interventions in three Kenyan districts, 2014. BMC Public Health. 2016;16:152. doi: 10.1186/s12889-016-2823-5 26880141 PMC4754928

[61] Ibraheem R, Akintola M, Abdulkadir M, Ameen H, Bolarinwa O, Adeboye M. Effects of call reminders, short message services (SMS) reminders, and SMS immunization facts on childhood routine vaccination timing and completion in Ilorin, Nigeria. Afr Health Sci. 2021;21(2):951–9. doi: 10.4314/ahs.v21i2.57 34795755 PMC8568234

[62] Kawakatsu Y, Oyeniyi Adesina A, Kadoi N, Aiga H. Cost-effectiveness of SMS appointment reminders in increasing vaccination uptake in Lagos, Nigeria: A multi-centered randomized controlled trial. Vaccine. 2020;38(42):6600–8. doi: 10.1016/j.vaccine.2020.07.075 32788139

[63] Oladepo O, Dipeolu IO, Oladunni O. Outcome of reminder text messages intervention on completion of routine immunization in rural areas, Nigeria. Health Promot Int. 2021;36(3):765–73. doi: 10.1093/heapro/daaa092 33057615 PMC8384379

[64] Sampson S, Adenipekun A, Atobatele S, Ayodeji O, Omeje O, Oni F. An assessment of the effectiveness of an electronic wristband in improving routine immunization timeliness and reducing drop-out. J Public Health (Oxf). 2023;45(4):947–56. doi: 10.1093/pubmed/fdad134 37553100 PMC10687606

[65] Schlumberger M, Bamoko A, Yaméogo TM, Rouvet F, Ouedraogo R, Traoré B, et al. Positive impact on the Expanded Program on Immunization when sending call-back SMS through a Computerized Immunization Register, Bobo Dioulasso (Burkina Faso). Bull Soc Pathol Exot. 2015;108(5):349–54. doi: 10.1007/s13149-015-0455-4 26498331

[66] Yunusa U, Ibrahim AH, Ladan MA, Gomaa HEM. Effect of mobile phone text message and call reminders in the completeness of pentavalent vaccines in Kano state, Nigeria. J Pediatr Nurs. 2022;64:e77–83. doi: 10.1016/j.pedn.2021.12.026 35042638

[67] Yunusa U, Garba SN, MacDonald SE, Bello UL, Ibrahim AH, Abdulrashid I, et al. Utilization of Mobile Reminders in Improving the Completeness and Timeliness of Routine Childhood Immunization in Kano Metropolis, Nigeria: A Randomized Controlled Trial. J Pediatr Health Care. 2024;38(5):727–36. doi: 10.1016/j.pedhc.2024.03.002 38551537

[68] Suffoletto B. Text Message Behavioral Interventions: From Here to Where? Curr Opin Psychol. 2016;9:16–21. doi: 10.1016/j.copsyc.2015.09.012 26665157 PMC4671292

[69] Currie GE, McLeod C, Waddington C, Snelling TL. SMS-based interventions for improving child and adolescent vaccine coverage and timeliness: a systematic review. BMC Public Health. 2024;24(1):1753. doi: 10.1186/s12889-024-18900-4 38956527 PMC11218178

[70] Islam FMA, Lambert EA, Islam SMS, Hosen MA, Thompson BR, Lambert GW. Understanding the sociodemographic factors associated with intention to receive SMS messages for health information in a rural area of Bangladesh. BMC Public Health. 2021;21(1):2326. doi: 10.1186/s12889-021-12418-9 34969382 PMC8719406

[71] Clark NM, Janz NK, Dodge JA, Mosca L, Lin X, Long Q, et al. The effect of patient choice of intervention on health outcomes. Contemp Clin Trials. 2008;29(5):679–86. doi: 10.1016/j.cct.2008.04.002 18515187 PMC2577598

[72] Sterne JA, Egger M, Moher D. Addressing Reporting Biases. Cochrane Handbook for Systematic Reviews of Interventions. Wiley. 2008:297–333. doi: 10.1002/9780470712184.ch10

[73] Wakadha H, Chandir S, Were EV, Rubin A, Obor D, Levine OS, et al. The feasibility of using mobile-phone based SMS reminders and conditional cash transfers to improve timely immunization in rural Kenya. Vaccine. 2013;31(6):987–93. doi: 10.1016/j.vaccine.2012.11.093 23246258 PMC4603391

[74] Bassani DG, Arora P, Wazny K, Gaffey MF, Lenters L, Bhutta ZA. Financial incentives and coverage of child health interventions: a systematic review and meta-analysis. BMC Public Health. 2013;13 Suppl 3(Suppl 3):S30. doi: 10.1186/1471-2458-13-S3-S30 24564520 PMC3847540

[75] Levine G, Salifu A, Mohammed I, Fink G. Mobile nudges and financial incentives to improve coverage of timely neonatal vaccination in rural areas (GEVaP trial): A 3-armed cluster randomized controlled trial in Northern Ghana. PLoS One. 2021;16(5):e0247485. doi: 10.1371/journal.pone.0247485 34010312 PMC8133473

[76] Perosky JE, Munro ML, Kay JL, Nyanplu A, Williams G, Andreatta PB, et al. Texting From the Bush: Data Collection Using SMS Text Messaging in Areas of Low Network Coverage From Low-Literacy Providers. J Health Commun. 2015;20(9):1052–9. doi: 10.1080/10810730.2015.1018607 26147537 PMC4699450

[77] Kazi AM, Carmichael J-L, Hapanna GW, Wangoo PG, Karanja S, Wanyama D, et al. Assessing Mobile Phone Access and Perceptions for Texting-Based mHealth Interventions Among Expectant Mothers and Child Caregivers in Remote Regions of Northern Kenya: A Survey-Based Descriptive Study. JMIR Public Health Surveill. 2017;3(1):e5. doi: 10.2196/publichealth.5386 28137702 PMC5306611

[78] Materia FT, Smyth JM, Puoane T, Tsolekile L, Goggin K, Kodish SR, et al. Implementing text-messaging to support and enhance delivery of health behavior change interventions in low- to middle-income countries: case study of the Lifestyle Africa intervention. BMC Public Health. 2023;23(1):1526. doi: 10.1186/s12889-023-16388-y 37563595 PMC10416414

[79] Worldbank. Cote D’Ivoire – Gender Data. https://genderdata.worldbank.org/en/economies/cote-d-ivoire. 2019. Accessed 2024 October.

[80] Osanyin GE, Bankethomas A, Oluwole EO, Odeseye AK, Afolabi BB. Effects of a mHealth voice messaging intervention on antenatal care utilisation at primary care level in Lagos, Nigeria: a cluster randomised trial. J Public Health Afr. 2022;13(3):2222. doi: 10.4081/jphia.2022.2222 36277941 PMC9585595

[81] Downs SM, Sackey J, Kalaj J, Smith S, Fanzo J. An mHealth voice messaging intervention to improve infant and young child feeding practices in Senegal. Matern Child Nutr. 2019;15(4):e12825. doi: 10.1111/mcn.12825 30950190 PMC6860071

[82] Crandall A. Kenyan farmers’ use of cell phones: Calling preferred over SMS. In: Proceedings of M4D 2012, New Delhi, India, 2012:119.

[83] Jacobson Vann JC, Jacobson RM, Coyne-Beasley T, Asafu-Adjei JK, Szilagyi PG. Patient reminder and recall interventions to improve immunization rates. Cochrane Database Syst Rev. 2018;1(1):CD003941. doi: 10.1002/14651858.CD003941.pub3 29342498 PMC6491344

[84] Gurol-Urganci I, de Jongh T, Vodopivec-Jamsek V, Atun R, Car J. Mobile phone messaging reminders for attendance at healthcare appointments. Cochrane Database Syst Rev. 2013;2013(12):CD007458. doi: 10.1002/14651858.CD007458.pub3 24310741 PMC6485985

[85] Jadczyk T, Wojakowski W, Tendera M, Henry TD, Egnaczyk G, Shreenivas S. Artificial Intelligence Can Improve Patient Management at the Time of a Pandemic: The Role of Voice Technology. J Med Internet Res. 2021;23(5):e22959. doi: 10.2196/22959 33999834 PMC8153030

[86] Siddiqi DA, Ali RF, Munir M, Shah MT, Khan AJ, Chandir S. Effect of vaccine reminder and tracker bracelets on routine childhood immunization coverage and timeliness in urban Pakistan (2017-18): a randomized controlled trial. BMC Public Health. 2020;20(1):1086. doi: 10.1186/s12889-020-09088-4 32652969 PMC7353686

[87] Hammershaimb EA, Berry AA. Pre-erythrocytic malaria vaccines: RTS,S, R21, and beyond. Expert Rev Vaccines. 2024;23(1):49–52. doi: 10.1080/14760584.2023.2292204 38095048

[88] Osoro CB, Ochodo E, Kwambai TK, Otieno JA, Were L, Sagam CK, et al. Policy uptake and implementation of the RTS,S/AS01 malaria vaccine in sub-Saharan African countries: status 2 years following the WHO recommendation. BMJ Glob Health. 2024;9(4):e014719. doi: 10.1136/bmjgh-2023-014719 38688566 PMC11085798

[89] Adedokun ST, Yaya S. Factors influencing mothers’ health care seeking behaviour for their children: evidence from 31 countries in sub-Saharan Africa. BMC Health Serv Res. 2020;20(1):842. doi: 10.1186/s12913-020-05683-8 32894107 PMC7487813

[90] Gebeyehu NA, Asmare Adela G, Dagnaw Tegegne K, Birhan Assfaw B. Vaccination dropout among children in Sub-Saharan Africa: Systematic review and meta-analysis. Hum Vaccin Immunother. 2022;18(7):2145821. doi: 10.1080/21645515.2022.2145821 36459433 PMC9762788

[91] Di Pietrantonj C, Rivetti A, Marchione P, Debalini MG, Demicheli V. Vaccines for measles, mumps, rubella, and varicella in children. Cochrane Database Syst Rev. 2021;11(11):CD004407. doi: 10.1002/14651858.CD004407.pub5 34806766 PMC8607336

[92] Mekonnen ZA, Gelaye KA, Were M, Tilahun B. Effect of Mobile Phone Text Message Reminders on the Completion and Timely Receipt of Routine Childhood Vaccinations: Superiority Randomized Controlled Trial in Northwest Ethiopia. JMIR Mhealth Uhealth. 2021;9(6):e27603. doi: 10.2196/27603 34128813 PMC8277338

[93] Kagucia EW, Ochieng B, Were J, Hayford K, Obor D, O’Brien KL, et al. Impact of mobile phone delivered reminders and unconditional incentives on measles-containing vaccine timeliness and coverage: a randomised controlled trial in western Kenya. BMJ Glob Health. 2021;6(1):e003357. doi: 10.1136/bmjgh-2020-003357 33509838 PMC7845730

[94] Oke GI, Sibomana O. Adoption of Digital Health Technology in Nigeria: A Scoping Review of Current Trends and Future Directions. Advances in Public Health. 2025;2025(1). doi: 10.1155/adph/4246285

[95] Muhammad F, Abdulkareem JH, Chowdhury AA. Major Public Health Problems in Nigeria: A review. SE Asia J Pub Health. 2017;7(1):6–11. doi: 10.3329/seajph.v7i1.34672

[96] Eze P, Agu SA, Agu UJ, Acharya Y. Acceptability of mobile-phone reminders for routine childhood vaccination appointments in Nigeria - a systematic review and meta-analysis. BMC Health Serv Res. 2021;21(1):1276. doi: 10.1186/s12913-021-07296-1 34836531 PMC8627092

[97] Akinrinade O, Ajayi I, Fatiregun A, Isere E, Yusuf BO. Ownership of mobile phones and willingness to receive childhood immunisation reminder messages among caregivers of infants in Ondo State, South-Western Nigeria. SAJCH South African Journal of Child Health. 2018;12:111–6. doi: 10.7196/SAJCH.2018.v12i3.1477

[98] Ibraheem RM, Akintola MA. Acceptability of Reminders for Immunization Appointments via Mobile Devices by Mothers in Ilorin, Nigeria: A Cross-sectional Study. Oman Med J. 2017;32(6):471–6. doi: 10.5001/omj.2017.91 29218123 PMC5702982

[99] Obi-Jeff C, Garcia C, Onuoha O, Adewumi F, David W, Bamiduro T, et al. Designing an SMS reminder intervention to improve vaccination uptake in Northern Nigeria: a qualitative study. BMC Health Serv Res. 2021;21(1):844. doi: 10.1186/s12913-021-06728-2 34416906 PMC8379866

[100] Bervell B, Al-Samarraie H. A comparative review of mobile health and electronic health utilization in sub-Saharan African countries. Soc Sci Med. 2019;232:1–16. doi: 10.1016/j.socscimed.2019.04.024 31035241

[101] Simmons EM, Singh K, Mpiima J, Kumar M, Weiss W. Assessing coverage of essential maternal and child health interventions using health-facility data in Uganda. Popul Health Metr. 2020;18(1):26. doi: 10.1186/s12963-020-00236-x 33036626 PMC7547522

[102] Ehlman DC, Magoola J, Tanifum P, Wallace AS, Behumbiize P, Mayanja R, et al. Evaluating a Mobile Phone-Delivered Text Message Reminder Intervention to Reduce Infant Vaccination Dropout in Arua, Uganda: Protocol for a Randomized Controlled Trial. JMIR Res Protoc. 2021;10(2):e17262. doi: 10.2196/17262 33625372 PMC7946592

[103] Namageyo-Funa A, Jalloh MF, Gleason B, Wallace AS, Friedman M, Sesay T, et al. Data on the implementation of VaxTrac electronic immunization registry in Sierra Leone. Data Brief. 2020;32:106167. doi: 10.1016/j.dib.2020.106167 32904335 PMC7452626

[104] Haskew J, Kenyi V, William J, Alum R, Puri A, Mostafa Y, et al. Use of Mobile Information Technology during Planning, Implementation and Evaluation of a Polio Campaign in South Sudan. PLoS One. 2015;10(8):e0135362. doi: 10.1371/journal.pone.0135362 26252383 PMC4529202

[105] Okano JT, Ponce J, Krönke M, Blower S. Lack of ownership of mobile phones could hinder the rollout of mHealth interventions in Africa. Elife. 2022;11:e79615. doi: 10.7554/eLife.79615 36255055 PMC9640192

[106] Duggal M, El Ayadi A, Duggal B, Reynolds N, Bascaran C. Editorial: Challenges in implementing digital health in public health settings in low and middle income countries. Front Public Health. 2023;10:1090303. doi: 10.3389/fpubh.2022.1090303 36703825 PMC9872111

[107] McCool J, Dobson R, Whittaker R, Paton C. Mobile Health (mHealth) in Low- and Middle-Income Countries. Annu Rev Public Health. 2022;43:525–39. doi: 10.1146/annurev-publhealth-052620-093850 34648368

