# Supplementary material for: Systematic review of mHealth and digital health interventions to improve childhood vaccination uptake in 19 Sub-Saharan African countries
Source: PLoS One. 2025 Dec 23;20(12):e0324117. doi: 10.1371/journal.pone.0324117 (PMC12725567; doi:10.1371/journal.pone.0324117)
Supplement: S2 File — (DOCX) [file pone.0324117.s002.docx]

**S2** – **PICOSS Framework and Inclusion/Exclusion Criteria**

**S2.1** The PICOSS framework used to guide this systematic review.

| **Aspect of PICOSS** | **The PICOSS used** |
| --- | --- |
| Population | Mothers/caregivers of children or infants (<5 years old) that are receiving the DTP or Pentavalent vaccines in the 19 SSA countries or HCWs that are involved in administering the DTP or Pentavalent vaccines. |
| Intervention | mHealth/DH interventions that are used in essential childhood immunisation programmes in SSA to increase vaccination uptake or coverage |
| Comparison | Non-digital strategies for increasing vaccination uptake in SSA (e.g. Physical or verbal appointment reminders). |
| Outcome | Vaccination Uptake or Coverage Rate (%), Vaccination Schedule Completion (%), Vaccination Schedule Timeliness Rate (%), Risk Ratio (RR) or Odds Ratio (OR) |
| Setting | 19 Countries in SSA (Benin, Burkina Faso, Burundi, Cameroon, Central African Republic, Chad, Cote d’Ivoire, Democratic Republic of Congo, Ghana, Guinea, Kenya, Liberia, Malawi, Mozambique, Niger, Nigeria, Sierra Leone, South Sudan and Uganda) |
| Study Design | Experimental Studies (Randomised Control Trials [RCTs] or Non-Randomised Control Trials [Non-RCTs]) or Pre-post case studies (Technology implementation or assessment study) |

**S2.2** PICOSS guided inclusion/exclusion criteria for assessing study inclusion eligibility.

| **PICOSS** | **Inclusion** | **Exclusion** |
| --- | --- | --- |
| Population | Any study with a study population receiving or administering the DTP or Pentavalent Vaccine. | Any studies that report results from other immunisation programmes for example, COVID-19 vaccines or Human Papillomavirus (HPV) |
| Intervention | Studies which implement an mHealth or Digital Health intervention to increase vaccination uptake. For example, automated-mobile phone appointment reminders. | Studies which implement non-digital interventions for increasing vaccination uptake. For example, physical or verbal appointment reminders. |
| Comparison | Non-digital interventions for increasing vaccine uptake (comparator arm for experimental studies) | Non-digital interventions are used to increase vaccine uptake in the immunisation programmes. |
| Outcome | Studies reporting vaccination uptake rate (%), schedule completion (%), vaccination timeliness rate (%), RR or OR. | N/A |
| Setting | 19 Countries in SSA rolling out malaria vaccine in 2024. | Other SSA countries that will not be rolling out malaria vaccine eg. South Africa |
| Study Design | Only epidemiological experimental studies (RCTs or Non-RCTs) or Technology implementation or assessment case studies. | Observational studies such as case-control or cross-sectional studies, other types of studies including SRs and RCT protocols. |
