# Supplementary material for: Systematic review of mHealth and digital health interventions to improve childhood vaccination uptake in 19 Sub-Saharan African countries
Source: PLoS One. 2025 Dec 23;20(12):e0324117. doi: 10.1371/journal.pone.0324117 (PMC12725567; doi:10.1371/journal.pone.0324117)
Supplement: S3 File — (DOCX) [file pone.0324117.s003.docx]

**S3 - Search Strategies**

**S3.1** The key search terms and four concept area clusters used to make up the search

| **Concept Area Clusters** | **Search Terms** |
| --- | --- |
| mHealth and Digital Health Interventions | digital health OR mhealth OR m-health OR ehealth OR e-health OR Cell Phone OR Smartphone OR mobile device OR laptop OR computer OR electronic OR technology OR telemedicine OR Communication technology OR Text Messaging OR Text* OR Short Message OR SMS OR mobile app* OR application OR software OR App* OR App-based OR Web OR Web-based OR Internet* OR Digital* OR WhatsApp OR social media |
| Childhood Immunisation Programmes | Vaccination* OR immunization* OR immunisation* OR vaccine* OR DTP vaccine OR Diphtheria-Pertussis-Tetanus vaccine OR Pentavalent vaccine OR Reminder System OR Appointment OR Scheduling |
| Outcomes (Vaccination Uptake) and Study Design | Uptake OR coverage OR completion OR Timeliness OR Trial OR Implementation OR intervention OR reminder OR appointment |
| Setting | Benin OR Burkina Faso OR Burundi OR Cameroon OR Central African Republic OR Chad OR Cote d’Ivoire OR Democratic Republic of Congo OR Ghana OR Guinea OR Kenya OR Liberia OR Malawi OR Mozambique OR Niger OR Nigeria OR Sierra Leone OR South Sudan OR Uganda |

**Grey Literature**

Grey Literature search comprised search sources OpenHIA (OpenHIA.org), OpenMRS (OpenMRS.org) and WHO’s mHealth/DH working group publications (www.who.int/teams/digital-health-and-innovation). Only basic single keyword searches were conducted on these websites and were only related to immunisations cluster (‘immunisation’ or ‘immunization’ or ‘vaccine’ or ‘vaccination’). Search conducted on 17^th^ January 2025.

**Africa Journals Online (AJOL)**

Searched on 17^th^ January 2025 = 95 results

vaccination* OR immunization* OR immunisation* OR vaccine*

AND digital health OR mhealth OR m-health OR ehealth OR e-health OR Cell Phone OR smartphone OR mobile device OR telemedicine OR communication OR Text Messaging OR Short Message OR sms OR mobile app* OR app* OR app-based OR web OR web-based OR internet* OR digital* OR whatsapp OR social media

**Africa Index Medicus (AIM)**

Searched on 17^th^ January 2025 = 0 results

digital health OR mhealth OR ehealth OR Cell Phone OR Smartphone OR mobile device OR telemedicine OR Communication OR Text Messaging OR Short Message OR SMS OR mobile app* OR application OR App* OR App-based OR Web OR Web-based OR Internet* OR WhatsApp OR social media

AND Cluster Vaccination* OR immunization* OR immunisation* OR vaccine* OR Reminder System* OR Appointment Schedul* OR DTP Vaccin* OR Diphtheria-Pertussis-Tetanus vaccin*

**Cochrane Central**

Searched on 17^th^ January 2025 = 496 results

#1 (digital health):ti,ab,kw

#2 (mHealth):ti,ab,kw

#3 (eHealth):ti,ab,kw

#4 (cell phone):ti,ab,kw

#5 (smart phone):ti,ab,kw

#6 (mobile device):ti,ab,kw

#7 (laptop):ti,ab,kw

#8 (computer):ti,ab,kw

#9 (electronic):ti,ab,kw

#10 (technology):ti,ab,kw

#11 (telemedicine):ti,ab,kw

#12 (Communication):ti,ab,kw

#13 (text messaging):ti,ab,kw

#14 (short message):ti,ab,kw

#15 (SMS):ti,ab,kw

#16 (Mobile App*):ti,ab,kw

#17 (application):ti,ab,kw

#18 (software):ti,ab,kw

#19 (App based):ti,ab,kw

#20 ("web"):ti,ab,kw

#21 (Web based):ti,ab,kw

#22 (internet):ti,ab,kw

#23 (social media):ti,ab,kw

#24 (digital):ti,ab,kw

#25 (WhatsApp):ti,ab,kw

#26 (Text*):ti,ab,kw

#27 (App*):ti,ab,kw

#28 {OR #1-#27}

#29 (Vaccination*):ti,ab,kw

#30 (Immunization*):ti,ab,kw

#31 (Immunisation*):ti,ab,kw

#32 (Vaccine*):ti,ab,kw

#33 (DTP vaccin*):ti,ab,kw

#34 (Diphtheria-Pertussis-Tetanus vaccin*):ti,ab,kw

#35 (Pentavalent Vaccin*):ti,ab,kw

#36 Reminder System*

#37 Appointment Schedul*

#38 {OR #29-#37}

#39 #28 AND #38

#40 (Uptake OR Coverage OR Completion OR Timeliness OR Trial OR Implementation OR Intervention):ti,ab,kw

#41 (Benin OR Burkina Faso OR Burundi OR Cameroon OR Central African Republic OR Chad OR Cote d’Ivoire OR Democratic Republic of Congo OR Ghana OR Guinea OR Kenya OR Liberia OR Malawi OR Mozambique OR Niger OR Nigeria OR Sierra Leone OR South Sudan OR Uganda):ti,ab,kw

#42 #39 AND #40 AND #41

**Embase (Ovid)**

Searched on 17^th^ January 2025 = 377 results

1. electronic medical record/ or medical information/ or digital health/ or Internet/

2. mobile phone/ or mobile application/ or telemedicine/ or mhealth.mp. or mobile health application/

3. smartphone/

4. microcomputer/ or laptop/

5. ehealth.mp. or telehealth/

6. medical electronics/

7. digital health technology/ or digital technology/

8. communication technology/ or telecommunication/

9. automation/ or text messaging/

10. social media/

11. WhatsApp.mp.

12. WhatsApp.mp.

13. 1 or 2 or 3 or 4 or 5 or 6 or 7 or 8 or 9 or 10 or 11 or 12

14. vaccination/ or vaccination coverage/

15. immunization/ or mass immunization/

16. Immunisation.mp.

17. vaccine hesitancy/ or vaccine/

18. DTP vaccine.mp. or diphtheria pertussis tetanus vaccine/

19. Pentavalent vaccin*.mp.

20. diphtheria pertussis tetanus Haemophilus influenzae type b hepatitis B vaccine/

21. reminder system/

22. appointment schedul*.mp. [mp=title, abstract, heading word, drug trade name, original title, device manufacturer, drug manufacturer, device trade name, keyword heading word, floating subheading word, candidate term word]

23. 14 or 15 or 16 or 17 or 18 or 19 or 20 or 21 or 22

24. (Benin or Burkina Faso or Burundi or Cameroon or Central African Republic or Chad or Cote d'Ivoire or Democratic Republic of Congo or Ghana or Guinea or Kenya or Liberia or Malawi or Mozambique or Niger or Nigeria or Sierra Leone or South Sudan or Uganda).mp. [mp=title, abstract, heading word, drug trade name, original title, device manufacturer, drug manufacturer, device trade name, keyword heading word, floating subheading word, candidate term word]

25. 13 and 23 and 24

**Global Health (Ovid)**

Searched on 17^th^ January 2025 = 149 results

1. digital health.mp. or digital technology.sh. or social media.sh.

2. (technology or mobile equipment).sh. or mHealth/ or communication.sh.

3. ehealth.mp. or e-health/

4. smartphone.mp. or mobile telephones/

5. cell phone.mp.

6. mobile applications.sh.

7. computers/

8. electronics/

9. telemedicine/ or telecommunications.sh.

10. text messaging/

11. internet/

12. social media/

13. WhatsApp.mp.

14. 1 or 2 or 3 or 4 or 5 or 6 or 7 or 8 or 9 or 10 or 11 or 12 or 13

15. vaccination/ or mass vaccination/

16. immunization/ or immunization programmes/

17. immunisation.mp.

18. (vaccines or diphtheria pertussis tetanus vaccines).sh.

19. pentavalent vaccin*.mp.

20. reminder system.mp.

21. appointment schedul*.mp. [mp=abstract, title, original title, broad terms, heading words, cabicodes words]

22. 15 or 16 or 17 or 18 or 19 or 20 or 21

23. (Benin or Burkina Faso or Burundi or Cameroon or Central African Republic or Chad or Cote d'Ivoire or Democratic Republic of Congo or Ghana or Guinea or Kenya or Liberia or Malawi or Mozambique or Niger or Nigeria or Sierra Leone or South Sudan or Uganda).mp. [mp=abstract, title, original title, broad terms, heading words, cabicodes words]

24. 14 and 22 and 23

**Medline (Ovid)**

Searched on 17^th^ January 2025 = 163 results

1. Digital Health/ or Internet/ or Mobile Applications/ or Telemedicine/

2. mHealth.mp.

3. ehealth.mp.

4. Cell Phone/ or Smartphone/

5. microcomputers/ or computers, handheld/ or minicomputers/

6. electronics/ or digital technology/ or electronics, medical/

7. communication/ or "cell phone use"/

8. Text Messaging/

9. Social Media/

10. WhatsApp.mp.

11. 1 or 2 or 3 or 4 or 5 or 6 or 7 or 8 or 9 or 10

12. Vaccination/ or Mass Vaccination/ or Vaccination Hesitancy/ or Vaccination Coverage/

13. Immunization Programs/ or Immunization/ or Immunization Schedule/

14. Immunisation.mp.

15. Vaccines/

16. Diphtheria-Tetanus-Pertussis Vaccine/

17. Pentavalent vaccine.mp.

18. "Appointments and Schedules"/ or Reminder Systems/

19. 12 or 13 or 14 or 15 or 16 or 17 or 18

20. (Benin or Burkina Faso or Burundi or Cameroon or Central African Republic or Chad or Cote d'Ivoire or Democratic Republic of Congo or Ghana or Guinea or Kenya or Liberia or Malawi or Mozambique or Niger or Nigeria or Sierra Leone or South Sudan or Uganda).mp. [mp=title, book title, abstract, original title, name of substance word, subject heading word, floating sub-heading word, keyword heading word, organism supplementary concept word, protocol supplementary concept word, rare disease supplementary concept word, unique identifier, synonyms, population supplementary concept word, anatomy supplementary concept word]

21. 11 and 19 and 20

**Scopus**

Searched on 17^th^ January 2025 = 2812 results

(TITLE-ABS-KEY(benin OR {burkina faso} OR burundi OR cameroon OR {central african republic} OR chad OR {cote d'ivoire} OR {democratic republic of congo} OR ghana OR guinea OR kenya OR liberia OR malawi OR mozambique OR niger OR nigeria OR {sierra leone} OR {south sudan} OR uganda))

AND (TITLE-ABS-KEY(uptake OR coverage OR completion OR timeliness OR trial OR implementation OR intervention))

AND (TITLE-ABS-KEY(Vaccination* OR immunization* OR immunisation* OR vaccine* OR {DTP vaccine} OR {Diphtheria-Pertussis-Tetanus vaccine} OR {Pentavalent vaccine} OR {Reminder System} OR "Appointment Schedul*" ))

AND (TITLE-ABS-KEY( {digital health} OR mhealth OR m-health OR ehealth OR e-health OR {Cell Phone} OR smartphone OR {mobile device} OR laptop OR computer OR electronic OR technology OR telemedicine OR communication OR {Text Messaging} OR text* OR {Short Message} OR sms OR "mobile app*" OR application OR software OR app* OR app-based OR message OR web OR web-based OR internet OR whatsapp OR {social media} ))

**Web of Science**

Searched on 17^th^ January 2025 = 2332 results

“digital health” OR mhealth OR m-health OR ehealth OR e-health OR “Cell Phone” OR Smartphone OR “mobile device” OR laptop OR computer OR electronic OR technology OR telemedicine OR Communication OR “Text Messaging” OR Text* OR “Short Message” OR SMS OR “mobile app*” OR application OR software OR App* OR App-based OR Message OR Web OR Web-based OR Internet* OR Digital* OR WhatsApp OR “social media”

AND Vaccination* OR immunization* OR immunisation* OR vaccine* OR “DTP vaccin*” OR “Diphtheria-Pertussis-Tetanus vaccin*” OR “Pentavalent vaccin*” OR “Reminder System*” OR “Appointment Schedul*”

AND Uptake OR coverage OR completion OR Timeliness OR Trial OR Implementation OR Intervention OR reminder OR appointment

AND Benin OR “Burkina Faso” OR Burundi OR Cameroon OR “Central African Republic” OR Chad OR “Cote d’Ivoire” OR “Democratic Republic of Congo” OR Ghana OR Guinea OR Kenya OR Liberia OR Malawi OR Mozambique OR Niger OR Nigeria OR “Sierra Leone” OR “South Sudan” OR Uganda
