# Supplementary material for: Systematic review of mHealth and digital health interventions to improve childhood vaccination uptake in 19 Sub-Saharan African countries
Source: PLoS One. 2025 Dec 23;20(12):e0324117. doi: 10.1371/journal.pone.0324117 (PMC12725567; doi:10.1371/journal.pone.0324117)
Supplement: S4 File — (DOCX) [file pone.0324117.s004.docx]

**S4 – Included Studies and Description of Intervention-Type Subgroups**

| **Author and Year** | **Location** | **Title** |
| --- | --- | --- |
| Brown and Oluwatosin, 2017 [54] | Nigeria | Feasibility of implementing a cellphone-based reminder/recall strategy to improve childhood routine immunization in a low-resource setting: a descriptive report |
| Brown *et al*, 2016 [55] | Nigeria | Effects of Community Health Nurse-Led Intervention on Childhood Routine Immunization Completion in Primary Health Care Centers in Ibadan, Nigeria |
| Dissieka *et al*, 2019 [56] | Cote D'Ivoire | Providing mothers with mobile phone message reminders increases childhood immunization and vitamin A supplementation coverage in Cote d'Ivoire: a randomized controlled trial. |
| Ekhaguere *et al*, 2019 [57] | Nigeria | Automated phone call and text reminders for childhood immunisations (PRIMM): a randomised controlled trial in Nigeria |
| Eze and Adeleye, 2015 [58] | Nigeria | Enhancing routine immunization performance using innovative technology in an urban area of Nigeria |
| Gibson *et al*, 2017 [59] | Kenya | Mobile phone-delivered reminders and incentives to improve childhood immunisation coverage and timeliness in Kenya (M-SIMU): a cluster randomised controlled trial. |
| Haji *et al*, 2016 [60] | Kenya | Reducing routine vaccination dropout rates: evaluating two interventions in three Kenyan districts, 2014 |
| Ibraheem *et al*, 2021 [61] | Nigeria | Effects of call reminders, short message services (SMS) reminders, and SMS immunization facts on childhood routine vaccination timing and completion in Ilorin, Nigeria. |
| Kawakatsu *et al*, 2020 [62] | Nigeria | Cost-effectiveness of SMS appointment reminders in increasing vaccination uptake in Lagos, Nigeria: A multi-centered randomized controlled trial. |
| Oladepo *et al*, 2020 [63] | Nigeria | Outcome of reminder text messages intervention on completion of routine immunization in rural areas, Nigeria. |
| Sampson *et al*, 2023 [64] | Nigeria | An assessment of the effectiveness of an electronic wristband in improving routine immunization timeliness and reducing drop-out |
| Schlumberger *et al*, 2015 [65] | Burkina Faso | Positive impact on the Expanded Program on Immunization when sending call-back SMS through a Computerized Immunization Register, Bobo Dioulasso (Burkina Faso) |
| Yunusa *et al*, 2022 [66] | Nigeria | Effect of mobile phone text message and call reminders in the completeness of pentavalent vaccines in Kano state, Nigeria. |
| Yunusa *et al*, 2024 [67] | Nigeria | Utilization of Mobile Reminders in Improving the Completeness and Timeliness of Routine Childhood Immunization in Kano Metropolis, Nigeria: A Randomized Controlled Trial |

**S4.1** The author and year, study location and title of the 14 included studies.

**S4.2** Description of mHealth/DH intervention-types subgroup and which studies are included in each.

| **mHealth/DH Intervention-type Subgroup Investigated** | **Definition** | **Which Included Studies Are Related to Each Category** |
| --- | --- | --- |
| SMS-Only Appointment Reminders | Interventions that only comprised SMS appointment reminders. For example, Ibraheem *et al* (2021) sent this SMS to participants: “Dear parent, your child is due for the next vaccines tomorrow. Please bring your child for vaccination at the hospital at 8 am. Thank you.” [61]. | Seven studies included SMS-only interventions: Eze and Adeleye (2015) [58], Gibson *et al* (2017) [59], Haji et al (2016) [60], Ibraheem et al (2021) [61], Kawakatsu et al (2020) [62], Schlumberger *et al* (2015) [65], Yunusa *et al* (2022) [66]. |
| ‘SMS-Plus’ Interventions | These are SMS appointment reminder messages that have been ‘enhanced’. Either in combination with small cash incentives or provided additional info such as educational messages about immunisations. | Three studies were classed as ‘SMS-Plus’ interventions: Gibson *et al* (2017) [59], Ibraheem *et al* (2021) [61], Oladepo *et al* (2020) [63]. Gibson *et al* (2017) combined SMS with 75KES (Group B) and 200KES (Group C). Participants in Oladepo *et al* (2020) and Group C in Ibraheem *et al* (2021), received educational SMS messages. |
| ‘SMS and/or Voice Messages or Phone Calls’ | This subgroup refers to SMS appointment reminders either in conjunction with a voice-based component (voice message or phone call), or participants were given the choice between receiving SMS or voice message (allowing recipients to hear reminder). | Three studies were included in this subgroup: Dissieka *et al* (2019) [58], Ekhaguere *et al* (2019) [57], Yunusa *et al* (2024) [67]. Dissieka *et al* (2019) offered the choice of receiving SMS or voice message reminders. Ekhaguere *et al* (2019) combined SMS and voice messages, and Yunusa *et al* (2024) combined SMS with phone calls. |
| ‘Phone Call-Only reminders’ | These involved mothers/caregivers being called to remind them about their child’s upcoming immunisations (solely voice-based). | Three studies were included in this subgroup: Brown and Oluwatosin (2017) [54], Brown *et al* (2016) [55], Ibraheem *et al* (2021) [61]. Brown and Oluwatosin (2017), Group A in Brown *et al* (2016) and Group A in Ibraheem *et al* (2021) solely investigated phone call reminders. Group C in Brown *et al* (2016) investigated phone call reminders combined with specialist vaccinator/HCW training (not mHealth) |
| ‘Wearable Electronic Immunisation Alert Wristband’ | This was a wearable electronic wristband worn by mother/caregivers; it was digitally programmed to flash in the lead up to the day of a child’s immunisation appointment. The flashing red light could be easily spotted in the mother/caregivers’ hands. It flashed five times throughout the intervention, at week 0, week 6 (Penta1), week 10 (Penta2), week 14 (Penta3) and 9months. | Only Sampson *et al* (2023) [64]. |
