# Supplementary material for: Systematic review of mHealth and digital health interventions to improve childhood vaccination uptake in 19 Sub-Saharan African countries
Source: PLoS One. 2025 Dec 23;20(12):e0324117. doi: 10.1371/journal.pone.0324117 (PMC12725567; doi:10.1371/journal.pone.0324117)
Supplement: S5 File — (DOCX) [file pone.0324117.s005.docx]

**S5 – GRADE Certainty of Evidence Assessment Explanations**

**S5.3** GRADE Certainty of evidence assessment for vaccination coverage

**S5.4** GRADE Certainty of evidence assessment for vaccination timeline
