# Supplementary figures and images for: Systematic review of mHealth and digital health interventions to improve childhood vaccination uptake in 19 Sub-Saharan African countries
Source: PLoS One. 2025 Dec 23;20(12):e0324117. doi: 10.1371/journal.pone.0324117 (PMC12725567; doi:10.1371/journal.pone.0324117)

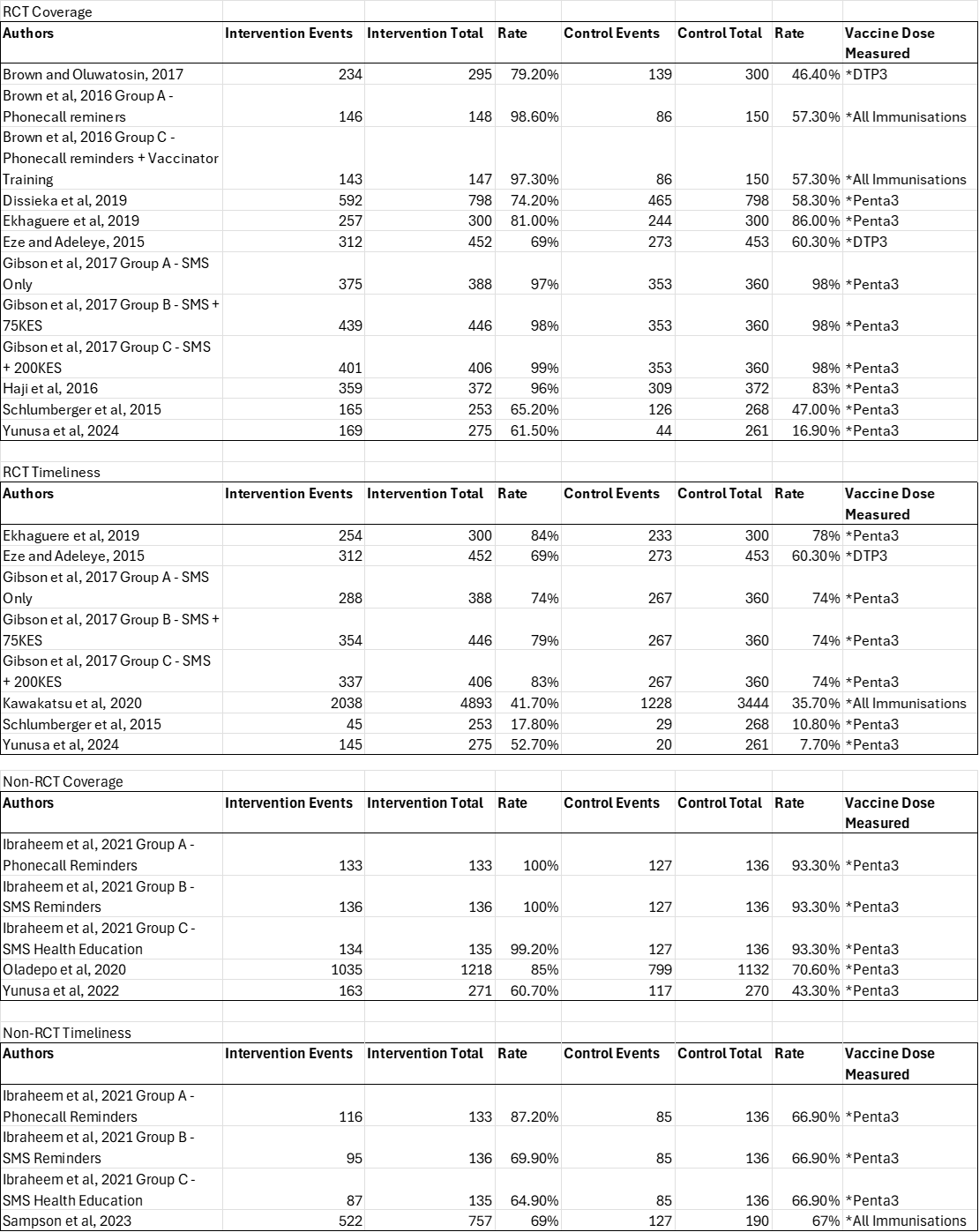
**S6 – Raw Quantitative Data used to calculate Forest Plots and LogOR**

Supplement: S6 File — (DOCX) [file pone.0324117.s006.docx]
